# Does Venous Drainage Route Matter? Portal Versus Systemic Drainage in Isolated Intestinal Transplantation: An Analysis of the National UNOS Database

**DOI:** 10.1111/petr.70400

**Published:** 2026-07-29

**Authors:** Vighnesh Venkatasamy, Amay Banker, Mahmoud Morsi, Gennaro Selvaggi, Akin Tekin, Rafael Miyashiro, Ahmad Nasrallah, Hiromichi Sato, Jennifer Garcia, Rodrigo Vianna

**Affiliations:** ^1^ Department of Surgery, Division of Transplant Surgery University of Miami/Jackson Health System Miami Florida USA; ^2^ Department of Pediatrics, Division of Gastroenterology, Hepatology and Nutrition University of Miami/Jackson Health System Miami Florida USA

**Keywords:** intestinal transplant, pediatric intestinal transplant, systemic versus portal drainage, venous drainage

## Abstract

**Background:**

Type of venous drainage in isolated intestinal transplantation (ITx) may influence immune responses through hepatic filtration of gut‐derived antigens and affect outcomes.

**Methods:**

We analyzed United Network for Organ Sharing data from 2013–2023 to compare portal versus systemic drainage in pediatric and adult ITx recipients. Graft and patient survival were assessed using Kaplan–Meier and Cox proportional hazards models, and acute rejection during the index admission was evaluated with multivariable logistic regression.

**Results:**

Among 480 recipients (137 pediatric, 343 adult), systemic drainage in pediatric patients was associated with higher acute rejection rates (50.7% vs. 32.4%, *p* = 0.029), and adjusted analysis confirmed systemic drainage as an independent risk factor (OR 2.32, 95% CI 1.00–5.37, *p* = 0.049). No survival differences were observed between drainage types in either age group.

**Conclusion:**

While venous drainage type did not affect long‐term graft or patient survival, portal drainage in pediatric ITx was associated with lower odds of early acute rejection, suggesting a potential immunologic benefit that warrants prospective validation.

## Graphical abstract

An analysis of UNOS registry data (2013–2023; *n* = 480) showed that portal venous drainage in isolated intestinal transplantation was associated with lower early acute rejection in pediatric recipients. Systemic and portal drainage showed comparable long‐term graft and patient survival in both pediatric and adult cohorts.

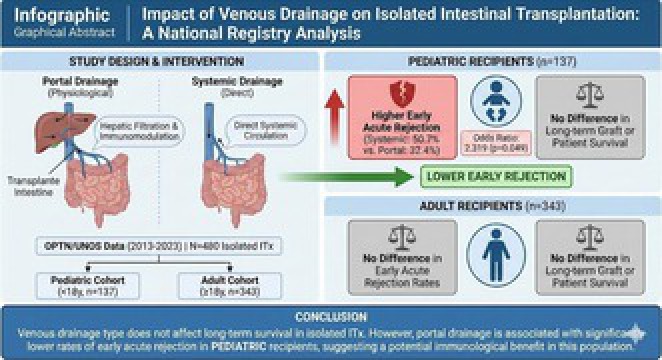

**Abbreviations**
BMI: body mass index
CI: confidence interval
CMV: Cytomegalovirus
HLA: Human Leukocyte Antigen
HR: hazard ratio
IQR: interquartile range
ITx: intestinal transplantation
LOS: length of stay
OPTN: Organ Procurement and Transplant Network
OR: odds ratio
STAR: Standard Transplant Analysis and Research
UNOS: United Network for Organ Sharing

## 1 Introduction

Intestinal transplantation (ITx) remains a valuable treatment option for patients with irreversible intestinal failure with complications from parenteral nutrition. Despite improving outcomes in recent years, ITx continues to present unique challenges as compared to other solid organ transplants, largely due to its high immunogenicity and constant exposure to enteric bacteria and antigens [1].

One technical aspect that may influence outcomes is the method of venous drainage used during the transplant. Surgeons typically choose between *portal drainage*, where venous return from the graft is routed through the liver (usually through anastomosis with the superior mesenteric vein), or *systemic drainage*, where outflow of the graft drains directly into the systemic circulation (typically inferior vena cava) [2]. Theoretically, routing blood through the liver allows hepatic Kupffer cells to filter out bacteria and other immune‐activating substances from the graft, potentially reducing the risk of rejection and infection [3]. On the other hand, systemic drainage is often favored for its technical simplicity, especially in patients with prior abdominal surgeries or complex anatomy. However, it may expose the recipient to higher levels of circulating endotoxins and pro‐inflammatory stimuli [4].

Preclinical models have suggested that portal venous outflow may promote immune tolerance and prolong graft survival, although findings have been inconsistent across studies [5, 6, 7, 8]. Some retrospective series—including those from established centers such as the University of Miami and the University of Pittsburgh—have found no clear differences in patient or graft survival based on drainage strategy [2, 9]. Berney and colleagues noted fewer pulmonary infections in patients with portal‐drained grafts, possibly due to more effective hepatic filtration. They also noted portal drainage to have pharmacokinetic implications, as it can affect tacrolimus metabolism, often requiring dose adjustments to maintain therapeutic levels [9].

Despite these insights, the best approach to venous drainage in ITx remains uncertain. Much of the available evidence is drawn from retrospective studies with small sample sizes, and the rarity of the procedure makes prospective trials difficult to conduct. As a result, the potential immunologic and clinical benefits of portal versus systemic drainage continue to be debated.

In this study, we analyzed the national data from the Organ Procurement and Transplant Network (OPTN), comparing portal and systemic venous drainage strategies in recipients of isolated ITx. By examining outcomes across both pediatric and adult populations, we hope to determine whether one strategy offers a clinical advantage. Understanding the impact of venous outflow configuration may help refine surgical decision‐making and improve long‐term results in this patient group.

## 2 Methods

### 2.1 Study Design and Cohort Selection

**FIGURE 1:**
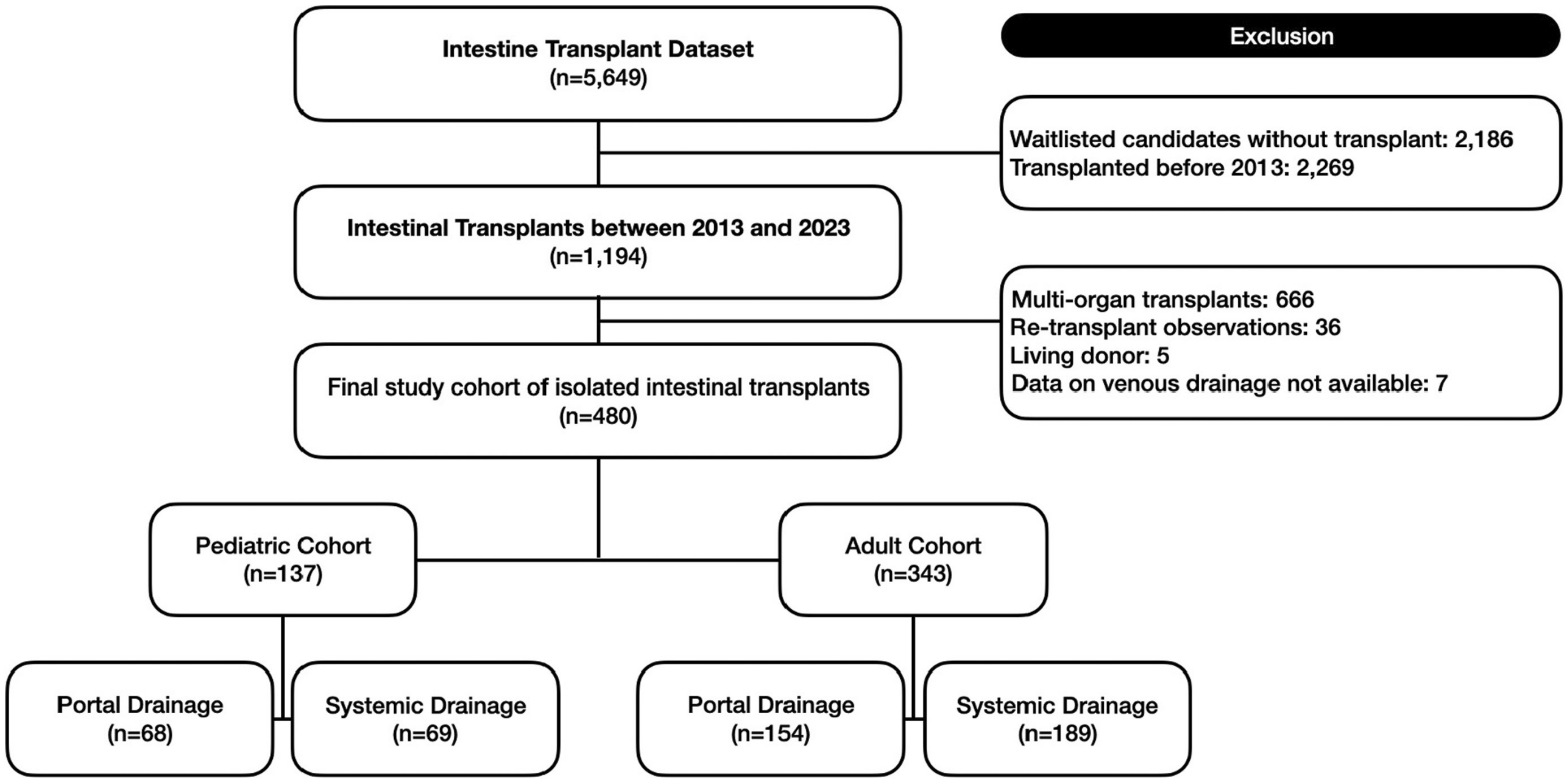
Methodology and study protocol.

This is a retrospective cohort study using the Standard Treatment Analysis and Research (STAR) files of the OPTN database dated 31st December 2023. Patients who underwent an isolated ITx between January 2013 and December 2023 were included in our cohort. Multi‐visceral transplants (*n* = 666), retransplanted observations (*n* = 36), and those receiving a living donor allograft (*n* = 5) were excluded from the cohort. Patients who were missing data on venous drainage (*n* = 7) were also removed from analysis. The cohort was then divided into a pediatric group (recipient age below 18 years) and an adult group (recipient age equal to or above 18 years). The cohorts were further subcategorized into a portal drainage group and a systemic drainage group based on the surgical technique used for the venous reconstruction in the recipient (Figure 1).

In the adult cohort, there were many instances in which a pediatric donor had donated to an adult recipient. So, the adult cohort was further subdivided into a pediatric‐to‐adult cohort and an adult‐to‐adult cohort based on the age of the donor for sensitivity analysis. This study did not require institutional review board approval because the UNOS dataset is limited to de‐identified patient data.

### 2.2 Data Collection

We identified isolated ITx using the UNOS STAR file named “INTESTINE_DATA.DTA” and then dropping all observations who were either on the waitlist without transplant and those with the variable “MULTI_ORG” marked as yes. Observations before the year 2013 were also dropped. Observations that had a previous transplant (variable PREV_TX) were also excluded.

The following donor covariates were examined: age, weight, body mass index (BMI), sex, race/ethnicity, mechanism of death, history of smoking, hypertension, inotropes requirement, donor blood cultures, and the relevant preoperative laboratory tests. Total ischemia time and human leukocyte antigen (HLA) mismatches at the A, B, and DR loci were examined as transplant‐related covariates. Recipient covariates included age, sex, race/ethnicity, diagnosis, weight, BMI, recent history of septicemia, cytomegalovirus (CMV) serology, location at time of transplant, and the relevant pre‐operative laboratory tests. Graft failure was defined as removal of the transplanted organ, death of the recipient, or placement of the recipient on chronic allograft support. High‐risk CMV status was defined as a donor with positive CMV serology and a recipient with negative CMV serology. Most variables in the dataset were complete, with minimal missing data. The primary outcome of interest was graft survival. Secondary outcomes included patient survival and acute rejection during the index transplant admission. The rejection outcome should also be interpreted cautiously because it was registry‐reported and limited to the index admission. The database does not provide histopathologic confirmation, rejection severity, timing of rejection, number of rejection episodes, treatment administered, or response to therapy.

### 2.3 Statistical Analysis

Data was first summarized using median and interquartile range for continuous variables and number and percentage for categorical variables. Wilcoxon *t*‐test was used for continuous variables and either the chi‐square test or Fisher exact test as appropriate was used for categorical variables to determine differences between group characteristics. Unadjusted graft and recipient survival within the two groups was evaluated using Kaplan–Meier survival analysis and log‐rank test. Adjusted graft and patient survival was evaluated using multivariable Cox proportional hazards models. The proportional hazards assumption was evaluated using scaled Schoenfeld residuals. Episodes of rejection within the index admission were evaluated with a multivariable logistic regression model. Covariates were selected a priori based on clinical relevance and prior literature, and all were included in the multivariable model to ensure appropriate adjustment for potential confounders. In a secondary analysis, we first ran univariable models for each factor and then built a multivariable model including only those variables with *p* < 0.10. All analyses were performed using STATA version 18.0 (Stata Statistical Software: Release 18. College Station, TX: StataCorp LLC).

## 3 Results

The final cohort consisted of 480 isolated ITx. There were 137 (28.5%) pediatric recipients, of which 133 received an organ from a pediatric donor, while 4 received an organ from an adult donor. In the pediatric cohort, 68 (49.6%) recipients underwent portal venous drainage, while 69 (51.4%) patients underwent systemic drainage. There were 343 (71.5%) adult recipients in the cohort, of which 154 (44.9%) patients underwent portal drainage, while 189 (55.1%) patients underwent systemic drainage. Within the adult cohort, 156 (45.5%) were adult‐to‐adult donations, and there were 187 (54.5%) instances when a pediatric donor donated to an adult recipient.

### 3.1 Donor Characteristics

In the pediatric cohort, the median donor age was 1 year, and the median donor weight was 12.9 kg for portal drainage while the median donor age was 2 years, and median donor weight was 13.3 kg for systemic drainage. No significant differences were found in pediatric donor characteristics between the two groups (Table 1).

**TABLE 1:** Donor demographics.

	Pediatric cohort		*p*	Adult cohort		*p*
	Portal *n = 68*	Systemic *n = 69*		Portal *n = 154*	Systemic *n = 189*	
Donor Age	1.0 (1.0,3.5)	2.0 (0.0,3.0)	0.809	18.0 (14.0,26.0)	16.0 (10.0,21.0)	0.003
Donor Sex						
Female	23 (33.8%)	31 (44.9%)	0.184	56 (36.4%)	65 (34.4%)	0.704
Male	45 (66.2%)	38 (55.1%)		98 (63.6%)	124 (65.6%)	
Donor Weight	12.9 (9.9, 17.7)	13.3 (9.5, 17.0)	0.582	59.0 (48.0, 68.0)	54.0 (39.9, 64.0)	0.005
Donor BMI	17.6 (15.5, 19.2)	16.6 (15.6, 18.8)	0.455	20.2 (18.2, 23.2)	20.9 (17.7, 22.9)	0.892
Donor Ethnicity						
White	36 (52.9%)	33 (47.8%)	0.154	102 (66.2%)	112 (59.3%)	0.292
African American	14 (20.6%)	25 (36.2%)		36 (23.4%)	44 (23.3%)	
Hispanic	15 (22.1%)	10 (14.5%)		14 (9.1%)	27 (14.3%)	
Other	3 (4.4%)	1 (1.4%)		2 (1.3%)	6 (3.2%)	
Inotropes at Procurement						
No	36 (52.9%)	42 (60.9%)	0.349	88 (57.1%)	97 (51.9%)	0.331
Yes	32 (47.1%)	27 (39.1%)		66 (42.9%)	90 (48.1%)	
Donor Hypertension						
No	68 (100%)	69 (100%)		151 (98.1%)	182 (96.3%)	0.521
Yes				3 (1.9%)	7 (3.7%)	
Donor Smoking						
No	68 (100%)	69 (100%)		151 (98.1%)	186 (98.4%)	1.000
Yes				3 (1.9%)	3 (1.6%)	
Donor Blood Infection						
No	58 (85.3%)	64 (92.8%)	0.182	144 (93.5%)	179 (94.7%)	0.651
Yes	10 (14.7%)	5 (7.2%)		10 (6.5%)	10 (5.3%)	
Mechanism of Death						
Cardiovascular	3 (4.4%)	3 (4.4%)	0.872	11 (7.1%)	13 (6.9%)	0.435
Trauma	29 (42.6%)	34 (50.0%)		94 (61.0%)	102 (54.0%)	
Anoxia	30 (44.1%)	26 (38.2%)		42 (27.3%)	67 (35.4%)	
Other	6 (8.8%)	5 (7.4%)		7 (4.5%)	7 (3.7%)	
Donor Labs						
Creatinine	0.2 (0.2, 0.4)	0.3 (0.2, 0.5)	0.071	0.7 (0.5, 1.0)	0.6 (0.5, 0.9)	0.162
Bilirubin	0.4 (0.3, 0.6)	0.4 (0.2, 0.7)	0.730	0.6 (0.4, 1.0)	0.6 (0.4, 0.9)	0.597
ALT	31.0 (19.5, 69.0)	28.0 (18.0, 39.0)	0.188	32.0 (19.0, 62.0)	33.5 (18.0, 54.0)	0.559
AST	44.0 (22.5, 95.0)	46.0 (27.0, 70.0)	0.923	41.5 (26.0, 69.0)	36.5 (22.0, 69.0)	0.466

*Note:* Data are presented as median (interquartile range) for continuous measures, and *n* (%) for categorical measures.

Abbreviations: ALT, Alanine Transaminase; AST, Aspartate Transaminase; BMI, Body Mass Index.

Among adult donors, the median age was 18 years for the portal drainage group and 16 years for the systemic drainage group (*p* = 0.003). The median donor weight was also significantly higher in the portal drainage group as compared to the systemic drained group (59 kg vs. 54 kg, p = 0.004). Trauma was the most common mechanism of death. All other characteristics were similar between the portal and systemic drainage groups in the adult cohort.

### 3.2 Recipient and Transplant Related Covariates

In the pediatric cohort, the median age was 5 years in the portal drainage group and 4 years in the systemic drainage group, with no significant differences in recipient characteristics between the two. Ethnic distribution, transplant location, and recent septicemia rates were similar across groups (Table 2).

**TABLE 2:** Recipient demographics.

	Pediatric to pediatric		*p*	Adult to adult		*p*
	Portal *n = 68*	Systemic *n = 69*		Portal *N* = 154	Systemic *N* = 189	
Recipient Age	5.0 (2.5, 9.5)	4.0 (2.0, 7.0)	0.256	40.0 (29.0, 53.0)	43.0 (32.0, 53.0)	0.440
Recipient Gender						
Female	29 (42.6%)	26 (37.7%)	0.553	91 (59.1%)	118 (62.4%)	0.528
Male	39 (57.4%)	43 (62.3%)		63 (40.9%)	71 (37.6%)	
Recipient Weight	17.6 (13.6, 27.3)	15.9 (12.6, 23.4)	0.326	63.0 (54.4, 76.7)	64.7 (53.4, 73.3)	0.890
Recipient BMI	17.1 (15.8, 19.4)	17.5 (15.7, 19.2)	0.973	22.7 (20.0, 25.3)	22.8 (20.2, 26.0)	0.723
Recipient Ethnicity						
White	35 (51.5%)	39 (56.5%)	0.818	120 (77.9%)	134 (70.9%)	0.343
African American	19 (27.9%)	15 (21.7%)		19 (12.3%)	24 (12.7%)	
Hispanic	10 (14.7%)	12 (17.4%)		11 (7.1%)	23 (12.2%)	
Other	4 (5.9%)	3 (4.3%)		4 (2.6%)	8 (4.2%)	
Location at Transplant						
Home	66 (97.1%)	66 (95.7%)	1.000	144 (93.5%)	177 (93.7%)	1.000
Hospitalized	2 (2.9%)	3 (4.3%)		10 (6.5%)	12 (6.3%)	
Recipient Recent Septicemia						
No	48 (71.6%)	48 (70.6%)	0.893	110 (72.4%)	140 (74.9%)	0.603
Yes	19 (28.4%)	20 (29.4%)		42 (27.6%)	47 (25.1%)	
Diagnosis						
Intestinal atresia/gastroschisis	22 (32.4%)	23 (33.3%)	0.604	3 (1.9%)	0 (0.0%)	0.050
Necrotizing Enterocolitis	10 (14.7%)	6 (8.7%)		3 (1.9%)	1 (0.5%)	
Intestinal Volvulus	7 (10.3%)	9 (13.0%)		12 (7.8%)	12 (6.3%)	
Resection for Crohn's/Tumor	0 (0.0%)	1 (1.4%)		26 (16.9%)	33 (17.5%)	
Mesenteric Thrombosis	0 (0.0%)	2 (2.9%)		16 (10.4%)	22 (11.6%)	
Functional Bowel Pathology	18 (26.5%)	14 (20.3%)		17 (11.0%)	8 (4.2%)	
Others (Unspecified)	11 (16.2%)	14 (20.3%)		77 (50.0%)	113 (59.8%)	
Recipient Labs						
Bilirubin	0.4 (0.3, 0.6)	0.4 (0.3, 0.6)	0.804	0.6 (0.3, 1.1)	0.6 (0.4, 1.2)	0.382
Creatinine	0.3 (0.2, 0.4)	0.3 (0.2, 0.5)	0.551	0.8 (0.7, 0.9)	0.8 (0.6, 1.0)	0.621
Albumin	3.7 (3.1, 4.0)	3.7 (3.2, 4.1)	0.674	3.7 (3.2, 4.1)	3.5 (3.1, 3.9)	0.011
Total Ischemia Time	6.5 (5.5, 7.5)	6.2 (5.3, 7.5)	0.516	6.4 (5.7, 7.5)	6.5 (5.5, 7.8)	0.779

*Note:* Data are presented as median (interquartile range) for continuous measures, and *n* (%) for categorical measures.

Abbreviations: ALT, Alanine Transaminase; AST, Aspartate Transaminase; BMI, body mass index.

In the adult cohort, the median age in the portal group was 40 years while in the systemic group median age was 43 years. Albumin levels were lower in the systemic drainage group among adults (3.5 vs. 3.7 g/dL, *p* = 0.011). The remaining parameters, including laboratory parameters such as bilirubin and creatinine levels, were comparable between drainage groups.

The most common diagnoses differed by age group, with intestinal atresia/gastroschisis being the most frequent in pediatric patients. Necrotizing enterocolitis was observed mainly in pediatric cases, whereas Crohn's disease and tumor‐related resections were seen more in adults.

Total ischemia time was similar between groups in both the adult and pediatric cohorts. HLA mismatch distribution was also similar between portal and systemic drainage groups in both pediatric and adult cohorts (Table S1). A‐locus mismatch rates were comparable across groups, though a trend toward more mismatches in the systemic drainage group was noted in adults (*p* = 0.093). B and DR locus mismatches were evenly distributed, with no significant differences between drainage types in either age group.

### 3.3 Outcomes

In pediatric patients, graft survival rates were 57.4% for the portal drainage group and 63.8% for systemic drainage (Table 3). Recipient survival was also comparable between the groups. In the pediatric cohort, acute rejection rates until discharge differed significantly, occurring more frequently in the systemic drainage group (50.7% vs. 32.4%, *p* = 0.029), while the median hospital length of stay (LOS) was significantly shorter in pediatric recipients with systemic drainage (42.0 vs. 53.5 days, *p* = 0.042). Only one patient in the pediatric cohort was lost to follow‐up.

**TABLE 3:** Outcomes.

	Pediatric to pediatric		*p*	Adult to adult		*p*
	Portal *n = 68*	Systemic *n = 69*		Portal *N* = 154	Systemic *N* = 189	
Graft Outcome						
Graft Survived	39 (57.4%)	44 (63.8%)	0.442	91 (59.1%)	97 (51.3%)	0.150
Graft Failed	29 (42.6%)	25 (36.2%)		63 (40.9%)	92 (48.7%)	
Recipient Outcome						
Recipient Alive	49 (72.1%)	52 (75.4%)	0.409	101 (65.6%)	111 (58.7%)	0.336
Recipient Mortality	9 (13.2%)	11 (15.9%)		43 (27.9%)	63 (33.3%)	
Lost to Follow‐Up	0 (0.0%)	1 (1.4%)		1 (0.6%)	0 (0.0%)	
Retransplanted	10 (14.7%)	5 (7.2%)		9 (5.8%)	15 (7.9%)	
Acute Rejection till discharge						
No	46 (67.6%)	34 (49.3%)	0.029	118 (77.1%)	150 (79.4%)	0.617
Yes	22 (32.4%)	35 (50.7%)		35 (22.9%)	39 (20.6%)	
Length of Stay	53.5 (34.0, 88.5)	42.0 (32.0, 60.0)	0.042	33.0 (23.0, 45.0)	32.5 (22.0, 52.0)	0.472

*Note:* Data are presented as median (interquartile range) for continuous measures, and *n* (%) for categorical measures.

Rejection in the index admission was further evaluated with multivariable logistic regression models (Table 4, Table S2). Recipients with systemic drainage demonstrated higher adjusted odds of developing rejection compared to those with portal drainage (OR 2.319, 95% CI 1.002–5.369, *p* = 0.049), although the borderline statistical significance and wide confidence interval warrant cautious interpretation. Additionally, Hispanic ethnicity in recipients was associated with increased rejection risk (OR = 3.763, *p* = 0.026). No associations were observed between rejection and donor/recipient sex, donor/recipient age, mismatches at B or DR loci, total ischemia time or CMV high‐risk status. We also performed a secondary analysis in which only variables significant in univariable models (*p* < 0.10) were included in the multivariable model, and the association between venous drainage and rejection remained significant in the pediatric cohort (Tables S3 and S4).

**TABLE 4:** Multivariable logistic regression model to evaluate predictors of rejection in the pediatric cohort.

	Odds ratio	95% Confidence Intervals		*p*
Venous Drainage				
Portal *(ref)*	—			
Systemic	2.319	1.002	5.369	0.049
HLA Mismatch (0–6)	0.883	0.308	2.535	0.817
A Locus Mismatch Level				
0 *(ref)*				
1	0.123	0.009	1.692	0.117
2	0.129	0.005	3.528	0.225
B Locus Mismatch Level				
1 *(ref)*	—			
2	1.528	0.342	6.833	0.579
DR Locus Mismatch Level				
0 *(ref)*	—			
1	0.790	0.224	2.778	0.713
2*	—	—	—	—
CMV High Risk				
No *(ref)*	—			
Yes	0.821	0.307	2.196	0.695
Donor Age	1.014	0.906	1.134	0.813
Recipient Age	0.996	0.875	1.134	0.950
Donor Sex				
Female *(ref)*	—			
Male	0.602	0.244	1.482	0.270
Recipient Sex				
Female *(ref)*	—			
Male	1.313	0.550	3.136	0.539
Ethnicity of recipient				
White	—			
African American	0.787	0.261	2.373	0.671
Hispanic	3.763	1.174	12.056	0.026
Other	1.280	0.210	7.794	0.789
Total Ischemia Time	0.917	0.739	1.138	0.430
Serum Albumin	0.606	0.293	1.251	0.176

Abbreviations: CMV, Cytomegalovirus; HLA, Human Leukocyte Antigen.

* Two DR mismatch omitted because of collinearity.

Adult graft survival rates were 59.1% for the portal drainage group and 51.3% for the systemic drainage group. The recipient survival rates were also similar between the groups. There was no difference in the rates of acute rejection or length of hospital stay in the adult cohort. Similar to the pediatric population, only one patient was lost to follow‐up in the adult cohort.

### 3.4 Survival Analysis

Survival analysis was performed using the log rank test and Kaplan Meier analysis at various time points—90 days, at 1 year, 5 years, and at 10 years post‐transplant to evaluate both short term and long‐term outcomes.

**FIGURE 2:**
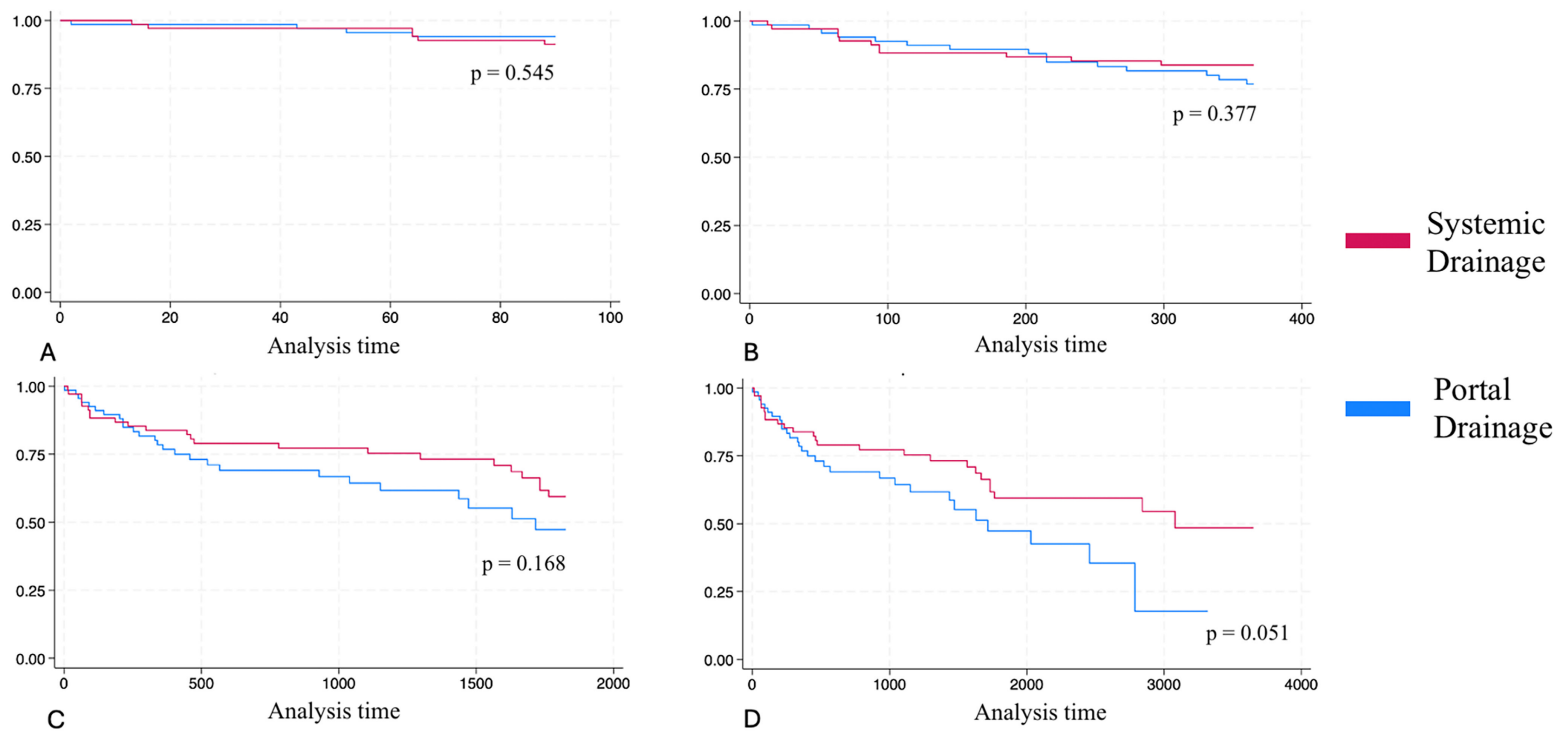
(A) Kaplan Meier curves for graft survival in the pediatric cohort at 90 days; (B) Kaplan Meier curves for graft survival in the pediatric cohort at 1 year; (C) Kaplan Meier curves for graft survival in the pediatric cohort at 5 years; (D) Kaplan Meier curves for graft survival in the pediatric cohort at 10 years.

In the pediatric cohort, graft survival did not differ significantly between the drainage strategies at 90 days, 1 year, or 5 years (Figure 2). Kaplan–Meier analysis suggested a trend toward improved 10‐year graft survival with systemic drainage (*p* = 0.051), but this was not seen in the multivariable Cox model (*p* = 0.152). In adjusted analysis, only total ischemia time was significantly associated with graft survival (Hazard Ratio (HR) = 1.13, 95% CI 1.00–1.28, *p* = 0.048) (Table S16). Patient survival was similar between groups at all time points between the two venous drainage strategies (Figure S1, Table S5).

**FIGURE 3:**
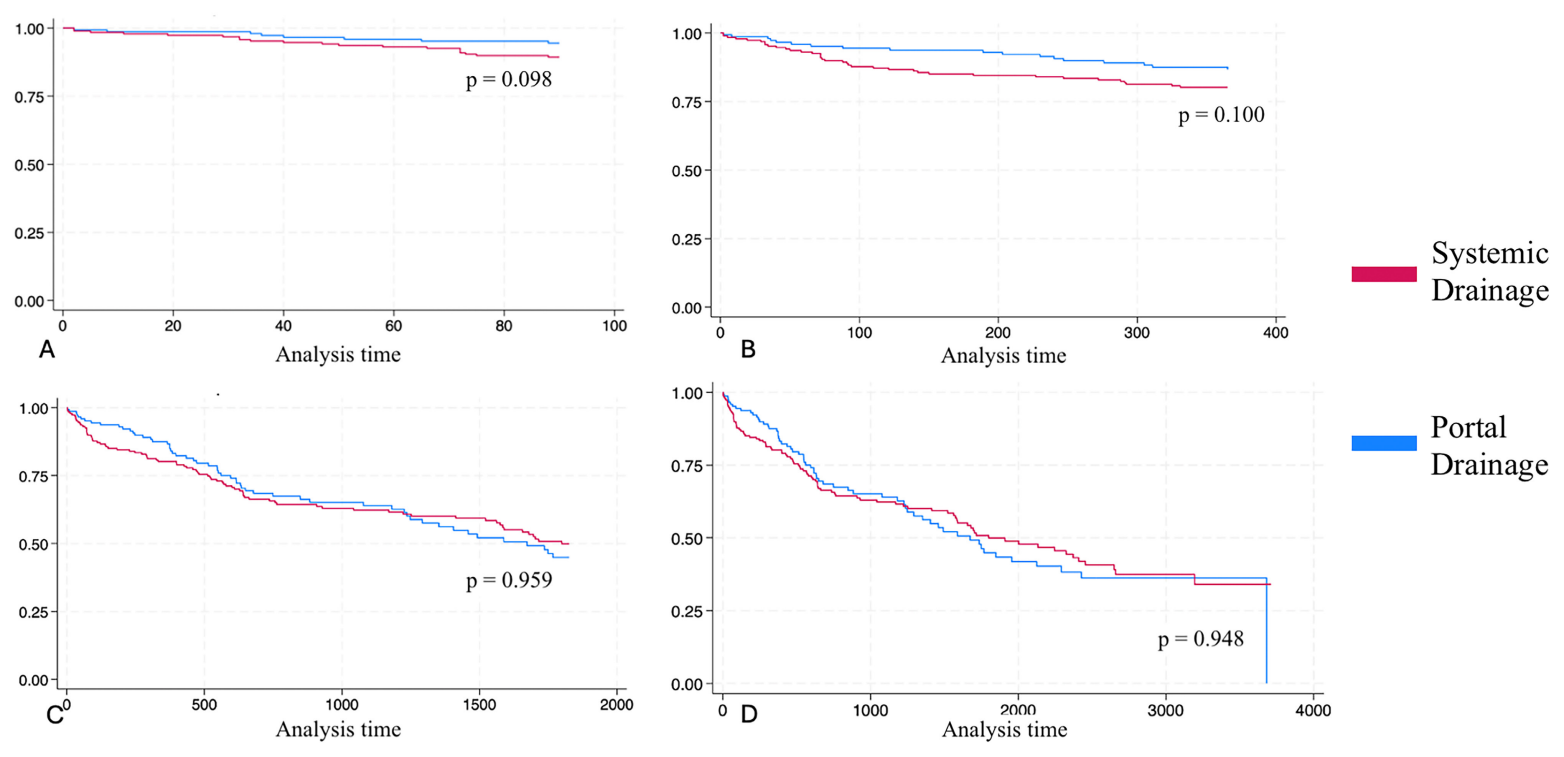
(A) Kaplan Meier curves for graft survival in the adult cohort at 90 days; (B) Kaplan Meier curves for graft survival in the adult cohort at 1 year; (C) Kaplan Meier curves for graft survival in the adult cohort at 5 years; (D) Kaplan Meier curves for graft survival in the adult cohort at 10 years.

In the adult cohort, no significant differences were observed in graft survival at 90 days, 1 year, 5 years, or overall, between the two groups in either unadjusted or adjusted analysis (Figure 3). In the adjusted analysis of the adult cohort, recipient serum albumin was the only variable significantly associated with graft survival (Table S6). Patient survival was also comparable across all time points between the two venous drainage strategies (Figure S2, Table S7).

### 3.5 Sensitivity Analysis

We also performed a sensitivity analysis stratifying adult recipients based on donor age (pediatric‐to‐adult vs. adult‐to‐adult transplants) (Tables S8–S10). In the adult‐to‐adult subgroup, graft survival appeared numerically higher in the portal drainage group (66% vs. 53%), though this did not reach statistical significance (*p* = 0.095) (Table S11). No association was found between early rejection and venous drainage strategies in this sub‐population (Table S12). Higher donor age, higher donor BMI, and lower recipient BMI were independently associated with poorer graft survival. Lower serum albumin and recent septicemia were also significant risk factors (Table S13).

The pediatric‐to‐adult subgroup had the lowest graft survival in this analysis (51.4% in portal drainage vs. 50.4% in systemic drainage). No association was found between early rejection and venous drainage strategies (Table S14), and no variable was significantly associated with graft failure in adjusted analysis for this cohort (Table S15).

## 4 Discussion

This study provides a comprehensive evaluation of the impact of venous drainage configuration on clinical outcomes in isolated ITx utilizing national registry data stratified by pediatric and adult recipients.

While no survival differences emerged between portal and systemic drainage in either age group, we identified a key pediatric‐specific finding: systemic venous drainage was associated with more than twice the odds of early acute rejection. This association should be considered exploratory and hypothesis‐generating, given the borderline statistical significance and the relatively wide confidence interval. Such an association was not observed in adults. One potential hypothesis is that developmental differences in immune regulation and hepatic antigen processing may contribute to differential immune responses in pediatric recipients; however, the present study was not designed to directly evaluate these mechanisms [9].

The pediatric immune system's developmental immaturity may render them particularly susceptible to immune activation in the early post‐transplant period. Therefore, routing the intestinal venous outflow through the liver may confer greater immunological benefits in this patient population. There is evidence supporting the liver's role as an immunomodulatory organ in transplantation [10, 11]. Prior studies have demonstrated that the liver filters gut‐derived antigens, endotoxins, and microbial products via Kupffer cells and hepatic antigen‐presenting cells [5, 12]. When intestinal effluent enters the liver via portal venous inflow, the immunogenic substances are intercepted leading to a complex local immune environment characterized by tolerance rather than immune activation. This process has been shown to attenuate systemic immune responses and facilitate antigen‐specific immune regulation, a mechanism supported by both experimental and clinical studies [5, 12, 13]. Prior investigations have demonstrated improved bacterial clearance and reduced systemic endotoxemia in portal‐drained ITx recipients [9].

The influence of hepatic metabolism in intestinal transplantation was also analyzed by Desai et al. who compared adult recipients of isolated ITx with those receiving a liver inclusive intestinal graft using OPTN registry data. They demonstrated superior graft survival and lower rejection rates of recipients receiving liver inclusive intestinal grafts compared to those undergoing isolated ITx. These findings were attributed, at least in part, to the immunomodulatory role of the liver [14]. Portal drainage theoretically offers similar immunologic benefits by routing intestinal effluent through the native liver. While our analysis echoed their finding of lower rates of early rejection in portal drained pediatric recipients, we did not note any survival advantage over systemic drainage. This discrepancy raises questions about the distinct mechanisms through which inclusion of liver in the graft confers a survival advantage and highlights a potential avenue for future research.

We acknowledge that a potential criticism of our hypothesis would be that the systemic drained venous blood ultimately reaches the hepatic sinusoids within a few circulatory cycles, regardless of initial drainage configuration. However, our objective was not to interrogate the physiology of hepatic immune surveillance, but rather to present clinical evidence from a large national cohort suggesting an increased incidence of rejection in pediatric recipients of systemically drained intestinal grafts. While the precise immunologic mechanisms underlying this association remain to be elucidated, our findings highlight the need for prospective, mechanistic studies to better understand the potential tolerogenic role of portal venous drainage in pediatric intestinal transplantation.

It is important to note that portal drainage can be technically demanding, particularly in re‐operative abdomens. In our cohort, pediatric recipients undergoing portal drainage had longer hospital stays. Portal drainage may require more demanding reconstruction, contributing to longer hospitalization. Conversely, the shorter length of stay in pediatric recipients with systemic drainage may reflect lower surgical complexity, or differences in institutional discharge practices., Because the registry does not capture timing, severity, treatment, or clinical consequences of rejection, we could not determine whether rejection episodes directly affected length of stay. Our findings are consistent with the experience reported by Colledan and colleagues, who have described the anatomical and technical demands of portal drainage, including the need for meticulous vascular alignment and tension‐free anastomoses to ensure graft viability [15]. Ultimately, the choice of venous drainage strategy must be individualized, considering recipient anatomy, surgical history, and institutional expertise [16, 17].

In our primary analysis, prolonged ischemia time in the pediatric cohort and lower recipient serum albumin in the adult cohort were the only factors significantly associated with graft and patient survival. The lowest graft survival was observed in pediatric‐to‐adult transplants, which may reflect the clinical frailty or cachectic status of recipients requiring smaller grafts. In the adult‐to‐adult subgroup analysis, higher donor BMI and lower recipient BMI were associated with poorer graft survival, suggesting outcomes could be influenced by donor‐recipient size mismatch. Advancing donor age and recent history of septicemia in recipients further compounded this risk, underscoring the importance of appropriate donor–recipient matching to mitigate graft failure.

This analysis is subject to several limitations inherent to retrospective studies utilizing registry data. The UNOS database does not capture the granular clinical details that may influence post‐transplant outcomes. Specifically, it lacks standardized data on immunosuppressive regimens, tacrolimus trough levels, histopathologic grading of rejection, timing of rejection episodes, and the occurrence or severity of infectious complications. These unmeasured variables may act as confounders and limit the interpretability of certain associations. Furthermore, the accuracy of venous drainage assignment relies on documentation by individual transplant centers, which may vary in completeness and could impact observed differences. The choice of venous drainage configuration may be influenced by intraoperative judgment, prior abdominal operations, vascular anatomy, technical complexity, or institutional preference, variables that are not comprehensively captured within the UNOS registry. Although our analyses utilized multivariable‐adjusted regression models incorporating clinically relevant donor, recipient, and transplant‐related covariates, residual confounding by indication remains possible. Accordingly, these findings should be interpreted as associative rather than causal.

## 5 Conclusions

This study represents one of the largest analyses examining the impact of venous drainage configuration on outcomes in isolated ITx. Venous drainage type did not affect long‐term graft or patient survival. Portal drainage in pediatric recipients was associated with lower adjusted odds of early acute rejection; however, this finding should be interpreted cautiously and considered hypothesis‐generating. Future prospective studies incorporating immune profiling, histopathologic evaluation, and pharmacokinetic data are warranted to validate these associations and to better identify the patients most likely to benefit from portal venous drainage as a modifiable surgical strategy.

## Author Contributions

V.V. participated in research design, writing of the paper, and data analysis. A.B. contributed to research design, writing of the paper, data analysis, and performance of the research. M.M. was involved in the writing of the manuscript and critical revision. G.S. contributed to research design and critical review of the paper. A.T. participated in research design and contributed to data analysis. R.M. was involved in the performance of the research and data analytics. A.N. contributed to the writing of the paper and data analysis. J.G. participated in the performance of the research, and revision of the manuscript. R.V. contributed to research design, writing of the paper, and data analysis, and provided overall supervision. All authors approved the final version of the manuscript.

## Supporting information


**Table S1:** Transplant Related Covariates.
**Table S2:** Multivariable Logistic regression model to evaluate predictors of rejection in the adult cohort.
**Table S3:** Stepwise Logistic regression model to evaluate predictors of rejection in the pediatric cohort.
**Table S4:** Step wise Logistic regression model to evaluate predictors of rejection in the adult cohort.
**Table S5:** Cox hazard model for adjusted patient survival analysis in the pediatric cohort.
**Table S6:** Cox hazard model for adjusted graft survival analysis in the adult cohort.
**Table S7:** Cox hazard model for adjusted patient survival analysis in the adult cohort.
**Table S8:** Donor Demographics in the adult cohort subdivided by age of donor.
**Table S9:** Recipient Demographics in the adult cohort subdivided by age of donor.
**Table S10:** Transplant related covariates in the adult cohort subdivided by age of donor.
**Table S11:** Outcomes in the adult cohort subdivided into type of donor.
**Table S12:** Multivariable Logistic regression model to evaluate predictors of rejection in the adult‐to‐adult cohort.
**Table S13:** Cox hazard model for adjusted graft survival analysis in the adult‐to‐adult cohort.
**Table S14:** Multivariable Logistic regression model to evaluate predictors of rejection in the pediatric‐to‐adult cohort.
**Table S15:** Cox hazard model for adjusted graft survival analysis in the pediatric‐to‐adult cohort.
**Table S16:** Cox hazard model* for adjusted graft survival analysis in the pediatric cohort.
**Figure S1:** Kaplan Meier curves for patient survival in the pediatric cohort at 90 days, 1, 5 and 10 years.
**Figure S2:** Kaplan Meier curves for patient survival in the adult cohort at 90 days, 1, 5 and 10 years.
**Figure S2A:** Kaplan Meier curves for patient survival in the adult cohort at 90 days, 2B: Kaplan Meier curves for patient survival in the adult cohort at 1 year. 2C: Kaplan Meier curves for patient survival in the adult cohort at 5 years and 2D: Kaplan Meier curves for patient survival in the adult cohort at 10 years.

## Data Availability

The data reported here have been supplied by the United Network for Organ Sharing as the contractor for the Organ Procurement and Transplantation Network.

## References

[1] M. Berger , A. Zeevi , D. G. Farmer , K. M. Abu‐Elmagd , and M. Berger , “Immunologic Challenges in Small Bowel Transplantation,” American Journal of Transplantation 12 (2012): S2–S8, 10.1111/j.1600-6143.2012.04332.x. 23181675

[2] W. H. Schraut , V. S. Abraham , and K. K. Lee , “Portal Versus Systemic Venous Drainage for Small‐Bowel Allografts,” Surgery 98, no. 3 (1985): 579–586, https://pubmed.ncbi.nlm.nih.gov/3875908/. 3875908

[3] M. P. Callery , T. Kamei , and M. W. Flye , “Kupffer Cell Blockade Inhibits Induction of Tolerance by the Portal Venous Route‐PubMed,” Transplantation 6, no. 47 (1989): 1092–1094, https://pubmed.ncbi.nlm.nih.gov/2734830/. 2734830

[4] W. Zheng , L. Yang , S. Jiang , et al., “Role of Kupffer Cells in Tolerance Induction After Liver Transplantation,” Frontiers in Cell and Development Biology 11 (2023): 1179077, 10.3389/FCELL.2023.1179077. PMC10435084 37601106

[5] L. E. Wrenshall , J. D. Ansite , P. M. Eckman , M. J. Heilman , R. B. Stevens , and D. E. R. Sutherland , “Modulation of Immune Responses After Portal Venous Injection of Antigen,” Transplantation 71, no. 7 (2001): 841–850, 10.1097/00007890-200104150-00004. 11349714

[6] L. Cicalese , P. Sileri , M. Green , K. Abu‐Elmagd , S. Kocoshis , and J. Reyes , “Bacterial Translocation in Clinical Intestinal Transplantation,” Transplantation 71, no. 10 (2001): 1414–1417, 10.1097/00007890-200105270-00010. 11391228

[7] D. Shaffer , T. Diflo , W. Love , G. H. A. Clowes , T. Maki , and A. P. Monaco , “Immunologic and Metabolic Effects of Caval Versus Portal Venous Drainage in Small‐Bowel Transplantation,” Surgery 104, no. 3 (1988): 518–524. 3261896

[8] F. Hernández , Y. Zou , G. López , et al., “Is Portal Venous Outflow Better Than Systemic Venous Outflow in Small Bowel Transplantation? Experimental Study in Syngeneic Rats,” Journal of Pediatric Surgery 40, no. 2 (2005): 336–340, 10.1016/j.jpedsurg.2004.10.016. 15750926

[9] T. Berney , T. Kato , S. Nishida , et al., “Portal Versus Systemic Drainage of Small Bowel Allografts: Comparative Assessment of Survival, Function, Rejection, and Bacterial Translocation,” Journal of the American College of Surgeons 195, no. 6 (2002): 804–813, 10.1016/S1072-7515(02)01482-5. 12495313

[10] N. Abrol , C. C. Jadlowiec , and T. Taner , “Revisiting the Liver's Role in Transplant Alloimmunity,” World Journal of Gastroenterology 25, no. 25 (2019): 3123, 10.3748/WJG.V25.I25.3123. 31333306 PMC6626728

[11] J. Y. Kim , K. H. Bin , J. M. Kim , et al., “Immunoprotective Effect of Liver Allograft on Patients With Combined Liver and Kidney Transplantation,” Annals of Transplantation 29 (2024): 942763, 10.12659/AOT.942763. PMC10858615 38319291

[12] G. V. Mazariegos , “Intestinal Transplantation: Current Outcomes and Opportunities,” Current Opinion in Organ Transplantation 14, no. 5 (2009): 515–521, 10.1097/MOT.0B013E328330680D. 19623070

[13] Y. Chen , C. R. Ong , G. J. McKenna , A. L. F. Mui , R. M. Smith , and S. W. Chung , “Induction of Immune Hyporesponsiveness After Portal Vein Immunization With Ovalbumin,” Surgery 129, no. 1 (2001): 66–75, 10.1067/MSY.2001.109059. 11150035

[14] C. S. Desai , A. C. Gruessner , K. M. Khan , et al., “Isolated Intestinal Transplants vs. Liver‐Intestinal Transplants in Adult Patients in the United States: 22 Yr of OPTN Data,” Clinical Transplantation 26, no. 4 (2012): 622–628, 10.1111/J.1399-0012.2011.01579.X. 22192061

[15] M. Colledan , C. Zanfi , and A. D. Pinna , “Technical Aspects of Intestinal Transplantation,” Current Opinion in Organ Transplantation 18, no. 3 (2013): 291–297, 10.1097/MOT.0B013E3283615DA1. 23665545

[16] Y. Avitzur and D. Grant , “Intestine Transplantation in Children: Update 2010,” Pediatric Clinics of North America 57, no. 2 (2010): 415–431, 10.1016/J.PCL.2010.01.019. 20371045

[17] G. Selvaggi and A. G. Tzakis , “Small Bowel Transplantation: Technical Advances/Updates,” Current Opinion in Organ Transplantation 14, no. 3 (2009): 262–266, 10.1097/MOT.0B013E328329CDC5. 19349867

