# Supplementary material for: Does Venous Drainage Route Matter? Portal Versus Systemic Drainage in Isolated Intestinal Transplantation: An Analysis of the National UNOS Database
Source: Pediatr Transplant. 2026 Jul 29;30(7):e70400. doi: 10.1111/petr.70400 (PMC13418771; doi:10.1111/petr.70400)
Supplement: Supplementary file 1 — Table S1: Transplant Related Covariates. Table S2: Multivariable Logistic regression model to evaluate predictors of rejection in the adult cohort. Table S3: Stepwise Logistic regression model to evaluate predictors of rejection in the pediatric cohort. Table S4: Step wise Logistic regression model to evaluate predictors of rejection in the adult cohort. Table S5: Cox hazard model for adjusted patient survival analysis in the pediatric cohort. Table S6: Cox hazard model for adjusted graft survival analysis in the adult cohort. Table S7: Cox hazard model for adjusted patient survival analysis in the adult cohort. Table S8: Donor Demographics in the adult cohort subdivided by age of donor. Table S9: Recipient Demographics in the adult cohort subdivided by age of donor. Table S10: Transplant related covariates in the adult cohort subdivided by age of donor. Table S11: Outcomes in the adult cohort subdivided into type of donor. Table S12: Multivariable Logistic regression model to evaluate predictors of rejection in the adult‐to‐adult cohort. Table S13: Cox hazard model for adjusted graft survival analysis in the adult‐to‐adult cohort. Table S14: Multivariable Logistic regression model to evaluate predictors of rejection in the pediatric‐to‐adult cohort. Table S15: Cox hazard model for adjusted graft survival analysis in the pediatric‐to‐adult cohort. Table S16: Cox hazard model* for adjusted graft survival analysis in the pediatric cohort. Figure S1: Kaplan Meier curves for patient survival in the pediatric cohort at 90 days, 1, 5 and 10 years. Figure S2: Kaplan Meier curves for patient survival in the adult cohort at 90 days, 1, 5 and 10 years. Figure S2A: Kaplan Meier curves for patient survival in the adult cohort at 90 days, 2B: Kaplan Meier curves for patient survival in the adult cohort at 1 year. 2C: Kaplan Meier curves for patient survival in the adult cohort at 5 years and 2D: Kaplan Meier curves for patient survival in the adult cohort at 10 years. [file PETR-30-e70400-s001.docx]

## Supplemental File

| **Supplemental Table 1: Transplant Related Covariates** | | | | | | |
| --- | --- | --- | --- | --- | --- | --- |
|  | **Pediatric to pediatric** | | **p** | **Adult to adult** | | **p** |
|  | **Portal** | **Systemic** |  | **Portal** | **Systemic** |  |
|  | ***n=68*** | ***n=69*** |  | **N=154** | **N=189** |  |
| HLA Mismatch |  |  | 0.998 |  |  | 0.848 |
| 0 | - | - |  | 1 (0.7%) | 0 (0.0%) |  |
| 1 | 1 (1.6%) | 0 (0.0%) |  | 1 (0.7%) | 3 (1.7%) |  |
| 2 | 2 (3.1%) | 1 (1.7%) |  | 7 (4.7%) | 7 (3.9%) |  |
| 3 | 5 (7.8%) | 4 (6.8%) |  | 21 (14.0%) | 20 (11.1%) |  |
| 4 | 13 (20.3%) | 13 (22.0%) |  | 33 (22.0%) | 47 (26.1%) |  |
| 5 | 28 (43.8%) | 28 (47.5%) |  | 55 (36.7%) | 66 (36.7%) |  |
| 6 | 15 (23.4%) | 13 (22.0%) |  | 32 (21.3%) | 37 (20.6%) |  |
| A Locus Mismatch |  |  | 0.439 |  |  | 0.093 |
| 0 | 4 (6.2%) | 1 (1.7%) |  | 12 (7.9%) | 10 (5.6%) |  |
| 1 | 28 (43.8%) | 24 (40.7%) |  | 60 (39.7%) | 93 (51.7%) |  |
| 2 | 32 (50.0%) | 34 (57.6%) |  | 79 (52.3%) | 77 (42.8%) |  |
| B Locus Mismatch |  |  | 0.681 |  |  | 0.552 |
| 0 | - | - |  | 7 (4.6%) | 5 (2.8%) |  |
| 1 | 15 (23.4%) | 16 (27.1%) |  | 39 (25.8%) | 43 (23.9%) |  |
| 2 | 49 (76.6%) | 43 (72.9%) |  | 105 (69.5%) | 132 (73.3%) |  |
| DR Locus Mismatch |  |  | 0.415 |  |  | 0.803 |
| 0 | 4 (6.2%) | 1 (1.7%) |  | 12 (8.0%) | 11 (6.1%) |  |
| 1 | 23 (35.9%) | 26 (44.1%) |  | 62 (41.3%) | 75 (41.7%) |  |
| 2 | 37 (57.8%) | 32 (54.2%) |  | 76 (50.7%) | 94 (52.2%) |  |
| **Abbreviations:** HLA, Human Leukocyte Antigen.  Data are presented as median (interquartile range) for continuous measures, and n (%) for categorical measures. | | | | | | |

| **Supplemental Table 2: Multivariable Logistic regression model** **to evaluate predictors of rejection in the** **adult cohort** | | | | |
| --- | --- | --- | --- | --- |
|  | **Odds Ratio** | **95% CI** | | **p-value** |
| Venous Drainage |  |  |  |  |
| Portal *(ref)* | --- |  |  |  |
| Systemic | 0.911 | 0.520 | 1.596 | 0.745 |
| HLA mismatch (0-6) | 1.161 | 0.596 | 2.260 | 0.661 |
| A Locus Mismatch Level |  |  |  |  |
| 0 *(ref)* | --- |  |  |  |
| 1 | 1.263 | 0.279 | 5.710 | 0.761 |
| 2 | 1.443 | 0.210 | 9.931 | 0.709 |
| B Locus Mismatch Level |  |  |  |  |
| 0 *(ref)* | --- |  |  |  |
| 1 | 1.044 | 0.160 | 6.812 | 0.964 |
| 2 | 0.965 | 0.102 | 9.162 | 0.976 |
| DR Locus Mismatch Level |  |  |  |  |
| 0 *(ref)* | --- |  |  |  |
| 1 | 1.814 | 0.840 | 3.916 | 0.129 |
| 2 | --- |  |  |  |
| CMV High Risk |  |  |  |  |
| No *(ref)* | --- |  |  |  |
| Yes | 0.457 | 0.181 | 1.154 | 0.098 |
| Donor Age | 1.018 | 0.988 | 1.049 | 0.237 |
| Recipient Age | 0.981 | 0.960 | 1.001 | 0.065 |
| Donor Sex |  |  |  |  |
| Female *(ref)* | --- |  |  |  |
| Male | 1.024 | 0.560 | 1.871 | 0.939 |
| Recipient Sex |  |  |  |  |
| Female *(ref)* | --- |  |  |  |
| Male | 1.246 | 0.711 | 2.182 | 0.442 |
| Ethnicity of recipient |  |  |  |  |
| White | --- |  |  |  |
| African American | 1.018 | 0.433 | 2.395 | 0.967 |
| Hispanic | 1.510 | 0.619 | 3.684 | 0.365 |
| Other | 1.159 | 0.285 | 4.712 | 0.837 |
| Total Ischemia Time | 0.971 | 0.838 | 1.124 | 0.691 |
| Serum Albumin | 1.124 | 0.707 | 1.786 | 0.621 |
| Abbreviations: CMV, Cytomegalovirus; HLA, Human Leukocyte Antigen  *Two DR mismatch omitted because of collinearity. | | | | |

| **Supplemental Table 3: Stepwise Logistic regression model** **to evaluate predictors of rejection in the pediatric cohort** | | | | | | | | |
| --- | --- | --- | --- | --- | --- | --- | --- | --- |
|  | **Univariable analysis** | | | | **Multivariable analysis*** | | | |
|  | **OR** | **95% CI** | | **P** | **OR** | **95% CI** | | **P** |
| Venous Drainage |  |  |  |  |  |  |  |  |
| Portal *(ref)* | --- | --- | --- | --- | --- | --- | --- | --- |
| Systemic | 2.152 | 1.076 | 4.306 | 0.030 | 2.110 | 1.034 | 4.307 | 0.040 |
| Donor Age | 0.985 | 0.920 | 1.056 | 0.676 |  |  |  |  |
| Donor Sex |  |  |  |  |  |  |  |  |
| Female *(ref)* | --- | --- | --- | --- |  |  |  |  |
| Male | 0.728 | 0.364 | 1.457 | 0.370 |  |  |  |  |
| HLA Mismatch Level | 0.878 | 0.614 | 1.254 | 0.474 |  |  |  |  |
| A Locus Mismatch |  |  |  |  |  |  |  |  |
| 0 | --- | --- | --- | --- |  |  |  |  |
| 1 | 0.417 | 0.064 | 2.715 | 0.360 |  |  |  |  |
| 2 | 0.381 | 0.059 | 2.443 | 0.309 |  |  |  |  |
| B Locus Mismatch |  |  |  |  |  |  |  |  |
| 1 | --- | --- | --- | --- |  |  |  |  |
| 2 | 0.972 | 0.421 | 2.244 | 0.947 |  |  |  |  |
| DR Locus Mismatch |  |  |  |  |  |  |  |  |
| 0 | --- | --- | --- | --- |  |  |  |  |
| 1 | 1.034 | 0.158 | 6.764 | 0.972 |  |  |  |  |
| 2 | 0.852 | 0.133 | 5.449 | 0.866 |  |  |  |  |
| Recipient Age | 0.979 | 0.906 | 1.058 | 0.590 |  |  |  |  |
| Recipient Sex |  |  |  |  |  |  |  |  |
| Female *(ref)* | --- | --- | --- | --- |  |  |  |  |
| Male | 1.117 | 0.558 | 2.237 | 0.755 |  |  |  |  |
| Recipient Ethnicity |  |  |  |  |  |  |  |  |
| White | --- | --- | --- | --- | --- | --- | --- | --- |
| African American | 0.499 | 0.205 | 1.217 | 0.127 | 0.522 | 0.211 | 1.289 | 0.158 |
| Hispanic | 2.427 | 0.908 | 6.492 | 0.077 | 2.468 | 0.907 | 6.714 | 0.077 |
| Others | 1.040 | 0.217 | 4.983 | 0.961 | 1.123 | 0.228 | 5.536 | 0.886 |
| CMV High Risk |  |  |  |  |  |  |  |  |
| No | --- | --- | --- | --- |  |  |  |  |
| Yes | 1.217 | 0.579 | 2.559 | 0.605 |  |  |  |  |
| Recipient Terminal Labs |  |  |  |  |  |  |  |  |
| Bilirubin | 1.214 | 0.669 | 2.204 | 0.523 |  |  |  |  |
| Creatinine | 0.738 | 0.240 | 2.271 | 0.597 |  |  |  |  |
| Albumin | 0.742 | 0.450 | 1.223 | 0.242 |  |  |  |  |
| Ischemia Time | 0.922 | 0.774 | 1.099 | 0.364 |  |  |  |  |
| **Abbreviations:** HLA, Human Leukocyte Antigen; CMV, Cytomegalovirus | | | | | | | | |

| **Supplemental Table 4: Step wise Logistic regression model** **to evaluate predictors of rejection in the** **adult cohort** | | | | | | | | |
| --- | --- | --- | --- | --- | --- | --- | --- | --- |
|  | **Univariable analysis** | | | | **Multivariable analysis*** | | | |
|  | **OR** | **95% CI** | | **P** | **OR** | **95% CI** | | **P** |
| Venous Drainage |  |  |  |  |  |  |  |  |
| Portal *(ref)* | --- |  |  |  |  |  |  |  |
| Systemic | 0.877 | 0.523 | 1.469 | 0.617 | 0.896 | 0.533 | 1.506 | 0.678 |
| Donor Age | 1.015 | 0.989 | 1.042 | 0.258 |  |  |  |  |
| Donor Sex |  |  |  |  |  |  |  |  |
| Female *(ref)* | --- |  |  |  |  |  |  |  |
| Male | 1.094 | 0.636 | 1.882 | 0.746 |  |  |  |  |
| HLA Mismatch Level | 1.096 | 0.872 | 1.377 | 0.433 |  |  |  |  |
| A Locus Mismatch |  |  |  |  |  |  |  |  |
| 0 | --- |  |  |  |  |  |  |  |
| 1 | 1.675 | 0.466 | 6.015 | 0.429 |  |  |  |  |
| 2 | 1.986 | 0.556 | 7.089 | 0.291 |  |  |  |  |
| B Locus Mismatch |  |  |  |  |  |  |  |  |
| 0 | --- |  |  |  |  |  |  |  |
| 1 | 1.212 | 0.241 | 6.085 | 0.815 |  |  |  |  |
| 2 | 1.484 | 0.315 | 6.977 | 0.618 |  |  |  |  |
| DR Locus Mismatch |  |  |  |  |  |  |  |  |
| 0 | --- |  |  |  |  |  |  |  |
| 1 | 2.173 | 0.606 | 7.791 | 0.233 |  |  |  |  |
| 2 | 1.583 | 0.443 | 5.662 | 0.480 |  |  |  |  |
| Recipient Age | 0.981 | 0.962 | 1.000 | 0.053 | 0.981 | 0.963 | 1.000 | 0.056 |
| Recipient Sex |  |  |  |  |  |  |  |  |
| Female *(ref)* | --- |  |  |  |  |  |  |  |
| Male | 1.240 | 0.736 | 2.089 | 0.419 |  |  |  |  |
| Recipient Ethnicity |  |  |  |  |  |  |  |  |
| White | --- |  |  |  |  |  |  |  |
| African American | 1.048 | 0.473 | 2.325 | 0.907 |  |  |  |  |
| Hispanic | 1.650 | 0.742 | 3.670 | 0.219 |  |  |  |  |
| Others | 1.980 | 0.574 | 6.836 | 0.280 |  |  |  |  |
| CMV High Risk |  |  |  |  |  |  |  |  |
| No | --- |  |  |  |  |  |  |  |
| Yes | 0.562 | 0.241 | 1.309 | 0.182 |  |  |  |  |
| Recipient Terminal Labs |  |  |  |  |  |  |  |  |
| Bilirubin | 1.059 | 0.853 | 1.314 | 0.602 |  |  |  |  |
| Creatinine | 0.685 | 0.313 | 1.499 | 0.344 |  |  |  |  |
| Albumin | 1.372 | 0.912 | 2.064 | 0.129 |  |  |  |  |
| **Abbreviations:** HLA, Human Leukocyte Antigen; CMV, Cytomegalovirus | | | | | | | | |

| **Supplemental Table 5: Cox hazard model for adjusted patient survival analysis in the pediatric cohort** | | | | |
| --- | --- | --- | --- | --- |
|  | **Hazard Ratio** | **95% Confidence Intervals** | | **p-value** |
| Venous Drainage |  |  |  |  |
| Portal *(ref)* | --- |  |  |  |
| Systemic | 1.593 | 0.497 | 5.101 | 0.433 |
| Donor Age | 0.851 | 0.701 | 1.031 | 0.100 |
| Donor Sex |  |  |  |  |
| Female *(ref)* | --- |  |  |  |
| Male | 0.796 | 0.270 | 2.349 | 0.680 |
| Donor body mass index | 0.857 | 0.715 | 1.028 | 0.096 |
| Recipient Age | 1.110 | 0.928 | 1.328 | 0.252 |
| Recipient Sex |  |  |  |  |
| Female *(ref)* | --- |  |  |  |
| Male | 0.350 | 0.122 | 1.004 | 0.051 |
| Recipient body mass index | 1.206 | 0.971 | 1.498 | 0.090 |
| Ethnicity of recipient |  |  |  |  |
| White | --- |  |  |  |
| African American | 2.527 | 0.705 | 9.060 | 0.155 |
| Hispanic | 1.227 | 0.262 | 5.751 | 0.795 |
| Other | 2.896 | 0.290 | 28.970 | 0.365 |
| Serum Albumin | 0.880 | 0.394 | 1.969 | 0.756 |
| Total Ischemia Time | 1.431 | 1.147 | 1.784 | 0.001 |
| HLA Mismatch | 0.902 | 0.515 | 1.579 | 0.717 |
| Location at Transplant |  |  |  |  |
| At home | --- |  |  |  |
| Hospitalised | 1.833 | 0.222 | 15.110 | 0.573 |
| Recent history of Septicemia |  |  |  |  |
| No | --- |  |  |  |
| Yes | 1.469 | 0.436 | 4.957 | 0.535 |
| Abbreviations: CMV, Cytomegalovirus | | | | |

| **Supplemental Table 6: Cox hazard model for adjusted graft survival analysis in the adult cohort** | | | | |
| --- | --- | --- | --- | --- |
|  | **Hazard Ratio** | **95% Confidence Intervals** | | **p-value** |
| Venous Drainage |  |  |  |  |
| Portal *(ref)* | --- |  |  |  |
| Systemic | 0.964 | 0.670 | 1.385 | 0.842 |
| Donor Age | 1.018 | 0.997 | 1.039 | 0.095 |
| Donor Sex |  |  |  |  |
| Female *(ref)* | --- |  |  |  |
| Male | 1.199 | 0.817 | 1.759 | 0.354 |
| Donor body mass index | 0.995 | 0.942 | 1.052 | 0.868 |
| Recipient Age | 1.008 | 0.994 | 1.022 | 0.270 |
| Recipient Sex |  |  |  |  |
| Female *(ref)* | --- |  |  |  |
| Male | 0.840 | 0.577 | 1.222 | 0.361 |
| Recipient body mass index | 0.959 | 0.916 | 1.003 | 0.069 |
| Ethnicity of recipient |  |  |  |  |
| White | --- |  |  |  |
| African American | 1.068 | 0.621 | 1.836 | 0.813 |
| Hispanic | 1.046 | 0.592 | 1.849 | 0.876 |
| Other | 0.763 | 0.235 | 2.476 | 0.652 |
| Serum Albumin | 0.695 | 0.520 | 0.928 | 0.014 |
| Total Ischemia Time | 1.003 | 0.923 | 1.091 | 0.939 |
| HLA Mismatch | 1.045 | 0.902 | 1.210 | 0.557 |
| Location at Transplant |  |  |  |  |
| At home | --- |  |  |  |
| Hospitalised | 0.833 | 0.419 | 1.658 | 0.604 |
| Recent history of Septicemia |  |  |  |  |
| No | --- |  |  |  |
| Yes | 1.376 | 0.924 | 2.049 | 0.116 |
| Abbreviations: CMV, Cytomegalovirus | | | | |

| **Supplemental Table 7: Cox hazard model for adjusted patient survival analysis in the adult cohort** | | | | |
| --- | --- | --- | --- | --- |
|  | **Hazard Ratio** | **95% Confidence Intervals** | | **p-value** |
| Venous Drainage |  |  |  |  |
| Portal *(ref)* | --- |  |  |  |
| Systemic | 0.918 | 0.605 | 1.392 | 0.688 |
| Donor Age | 1.018 | 0.995 | 1.041 | 0.118 |
| Donor Sex |  |  |  |  |
| Female *(ref)* | --- |  |  |  |
| Male | 1.328 | 0.851 | 2.071 | 0.212 |
| Donor body mass index | 0.953 | 0.894 | 1.017 | 0.149 |
| Recipient Age | 1.032 | 1.015 | 1.050 | 0.000 |
| Recipient Sex |  |  |  |  |
| Female *(ref)* | --- |  |  |  |
| Male | 0.689 | 0.434 | 1.093 | 0.113 |
| Recipient body mass index | 0.964 | 0.914 | 1.017 | 0.181 |
| Ethnicity of recipient |  |  |  |  |
| White | --- |  |  |  |
| African American | 0.685 | 0.336 | 1.397 | 0.298 |
| Hispanic | 1.060 | 0.533 | 2.110 | 0.868 |
| Other | 0.599 | 0.143 | 2.510 | 0.483 |
| Serum Albumin | 0.670 | 0.476 | 0.942 | 0.021 |
| Total Ischemia Time | 1.008 | 0.918 | 1.106 | 0.871 |
| HLA Mismatch | 1.058 | 0.893 | 1.253 | 0.515 |
| Location at Transplant |  |  |  |  |
| At home | --- |  |  |  |
| Hospitalised | 0.779 | 0.328 | 1.854 | 0.573 |
| Recent history of Septicemia |  |  |  |  |
| No | --- |  |  |  |
| Yes | 1.237 | 0.769 | 1.991 | 0.381 |
| Abbreviations: HLA, Human Leukocyte Antigen; CMV, Cytomegalovirus | | | | |

| **Supplemental Table 8: Donor Demographics in the adult cohort subdivided by age of donor** | | | | | | |
| --- | --- | --- | --- | --- | --- | --- |
|  | **Pediatric to Adult** | | **p** | **Adult to Adult** | | **p** |
|  | **Portal** | **System** |  | **Portal** | **Systemic** |  |
|  | *n=72* | *n=115* |  | *n=82* | *n=74* |  |
| Donor Age | 13.0 (9.0, 15.0) | 11.0 (8.0, 15.0) | 0.139 | 25.0 (20.0, 29.0) | 22.0 (19.0, 31.0) | 0.563 |
| Donor Sex |  |  | 0.886 |  |  | 0.957 |
| Female | 22 (30.6%) | 34 (29.6%) |  | 34 (41%) | 31 (42%) |  |
| Male | 50 (69.4%) | 81 (70.4%) |  | 48 (59%) | 43 (58%) |  |
| Donor Weight | 58.9 (53.5, 71.6) | 62.1 (51.3, 72.0) | 0.735 | 65.0 (57.6, 71.0) | 63.0 (56.7, 70.0) | 0.370 |
| Donor BMI | 18.6 (16.7, 21.5) | 19.7 (16.5, 22.0) | 0.633 | 21.8 (19.3, 24.1) | 21.8 (20.4, 23.7) | 0.486 |
| Donor Ethnicity |  |  | 0.605 |  |  | 0.379 |
| White | 44 (61.1%) | 69 (60.0%) |  | 58 (71%) | 43 (58%) |  |
| African American | 20 (27.8%) | 26 (22.6%) |  | 16 (20%) | 18 (24%) |  |
| Hispanic | 7 (9.7%) | 16 (13.9%) |  | 7 (9%) | 11 (15%) |  |
| Other | 1 (1.4%) | 4 (3.5%) |  | 1 (1%) | 2 (3%) |  |
| Inotropes at Procurement |  |  | 0.763 |  |  | 0.156 |
| No | 35 (48.6%) | 58 (50.9%) |  | 53 (65%) | 39 (53%) |  |
| Yes | 37 (51.4%) | 56 (49.1%) |  | 29 (35%) | 34 (47%) |  |
| Donor Hypertension |  |  | 0.428 |  |  | 0.310 |
| No | 72 (100.0%) | 114 (99.1%) |  | 79 (96%) | 68 (92%) |  |
| Yes | 0 (0.0%) | 1 (0.9%) |  | 3 (4%) | 6 (8%) |  |
| Donor Smoking |  |  |  |  |  | 1.000 |
| No | 72 (100.0%) | 115 (100.0%) |  | 79 (96%) | 71 (96%) |  |
| Yes |  |  |  | 3 (4%) | 3 (4%) |  |
| Donor Blood Infection |  |  | 0.920 |  |  | 0.749 |
| No | 68 (94.4%) | 109 (94.8%) |  | 76 (93%) | 70 (95%) |  |
| Yes | 4 (5.6%) | 6 (5.2%) |  | 6 (7%) | 4 (5%) |  |
| Mechanism of Death |  |  | 0.137 |  |  | 0.157 |
| Cardiovascular | 8 (11.1%) | 8 (7.0%) |  | 3 (4%) | 5 (7%) |  |
| Trauma | 44 (61.1%) | 56 (48.7%) |  | 50 (61%) | 46 (62%) |  |
| Anoxia | 18 (25.0%) | 44 (38.3%) |  | 24 (29%) | 23 (31%) |  |
| Other | 2 (2.8%) | 7 (6.1%) |  | 5 (6%) | 0 (0%) |  |
| Donor Labs |  |  |  |  |  |  |
| Creatinine | 0.6 (0.4, 0.8) | 0.5 (0.4, 0.7) | 0.569 | 0.8 (0.7, 1.1) | 0.8 (0.7, 1.1) | 0.977 |
| Bilirubin | 0.6 (0.4, 0.9) | 0.5 (0.3, 0.8) | 0.121 | 0.6 (0.4, 1.0) | 0.7 (0.5, 1.0) | 0.135 |
| ALT | 31.5 (18.5, 61.0) | 36.0 (22.0, 62.0) | 0.609 | 32.5 (19.0, 64.0) | 25.0 (17.0, 44.0) | 0.084 |
| AST | 44.0 (26.0, 79.0) | 45.5 (27.0, 91.0) | 0.450 | 40.0 (27.0, 68.0) | 28.0 (20.0, 50.0) | 0.009 |
| **Abbreviations**: BMI, Body Mass Index; ALT, Alanine Transaminase; AST, Aspartate Transaminase  Data are presented as median (interquartile range) for continuous measures, and n (%) for categorical measures. | | | | | | |

| **Supplemental Table 9: Recipient Demographics in the adult cohort subdivided by age of donor** | | | | | | |
| --- | --- | --- | --- | --- | --- | --- |
|  | **Pediatric to Adult** | |  | **Adult to adult** | |  |
|  | **Portal** | **Systemic** | **p** | **Portal** | **Systemic** | **p** |
|  | *n=72* | *n=115* |  | *n=82* | *n=74* |  |
| Recipient Age | 37.5 (24.0, 50.5) | 43.0 (31.0, 53.0) | 0.084 | 44.0 (34.0, 55.0) | 43.5 (33.0, 53.0) | 0.772 |
| Recipient Gender |  |  | 0.759 |  |  | 0.813 |
| Female | 46 (63.9%) | 76 (66.1%) |  | 45 (55%) | 42 (57%) |  |
| Male | 26 (36.1%) | 39 (33.9%) |  | 37 (45%) | 32 (43%) |  |
| Recipient Weight | 58.9 (53.5, 71.6) | 62.1 (51.3, 72.0) | 0.735 | 66.0 (55.6, 81.2) | 67.3 (58.1, 75.1) | 0.875 |
| Recipient BMI | 22.5 (20.0, 24.7) | 21.7 (19.7, 25.3) | 0.980 | 23.3 (20.0, 27.0) | 23.7 (21.4, 26.6) | 0.333 |
| Recipient Ethnicity |  |  | 0.342 |  |  | 0.433 |
| White | 55 (76.4%) | 79 (68.7%) |  | 65 (79%) | 55 (74%) |  |
| African American | 10 (13.9%) | 17 (14.8%) |  | 9 (11%) | 7 (9%) |  |
| Hispanic | 7 (9.7%) | 14 (12.2%) |  | 4 (5%) | 9 (12%) |  |
| Other | 0 (0.0%) | 5 (4.3%) |  | 4 (5%) | 3 (4%) |  |
| Location at Transplant |  |  | 0.541 |  |  | 0.519 |
| Home | 66 (91.7%) | 109 (94.8%) |  | 78 (95%) | 68 (92%) |  |
| Hospitalised | 6 (8.3%) | 6 (5.2%) |  | 4 (5%) | 6 (8%) |  |
| Recipient Recent Septicemia |  |  | 0.072 |  |  | 0.285 |
| No | 46 (64.8%) | 87 (77.0%) |  | 64 (79%) | 53 (72%) |  |
| Yes | 25 (35.2%) | 26 (23.0%) |  | 17 (21%) | 21 (28%) |  |
| Diagnosis |  |  | 0.016 |  |  | 0.634 |
| Intestinal atresia / gastroschisis | 3 (4.2%) | 0 (0.0%) |  |  |  |  |
| Necrotizing Enterocolitis | 0 (0.0%) | 1 (0.9%) |  | 3 (4%) | 0 (0%) |  |
| Intestinal Volvulus | 7 (9.7%) | 6 (5.2%) |  | 5 (6%) | 6 (8%) |  |
| Resection for Crohn’s / Tumor | 12 (16.7%) | 21 (18.3%) |  | 14 (17%) | 12 (16%) |  |
| Mesenteric Thrombosis | 7 (9.7%) | 15 (13.0%) |  | 9 (11%) | 7 (9%) |  |
| Functional Bowel Pathology | 10 (13.9%) | 4 (3.5%) |  | 7 (9%) | 4 (5%) |  |
| Others (Unspecified) | 33 (45.8%) | 68 (59.1%) |  | 44 (54%) | 45 (61%) |  |
| Recipient Labs |  |  |  |  |  |  |
| Bilirubin | 0.5 (0.3, 1.0) | 0.6 (0.4, 1.2) | 0.244 | 0.6 (0.4, 1.1) | 0.7 (0.4, 1.1) | 0.810 |
| Creatinine | 0.8 (0.6, 0.9) | 0.7 (0.6, 1.0) | 0.875 | 0.8 (0.7, 1.0) | 0.8 (0.6, 1.1) | 0.830 |
| Albumin | 3.7 (3.2, 4.1) | 3.5 (3.0, 3.9) | 0.029 | 3.7 (3.2, 4.1) | 3.6 (3.3, 3.8) | 0.242 |
| Total Ischemia Time | 6.4 (5.5, 7.8) | 6.9 (6.0, 8.0) | 0.199 | 6.4 (5.7, 7.5) | 6.1 (5.1, 7.3) | 0.077 |
| **Abbreviations**: BMI, Body Mass Index; ALT, Alanine Transaminase; AST, Aspartate Transaminase  Data are presented as median (interquartile range) for continuous measures, and n (%) for categorical measures. | | | | | | |

| **Supplemental Table 10: Transplant related covariates in the adult cohort subdivided by age of donor** | | | | | | |
| --- | --- | --- | --- | --- | --- | --- |
|  | **Pediatric to Adult** | | **p** | **Adult to adult** | | **p** |
|  | **Portal** | **Systemic** |  | **Portal** | **Systemic** |  |
|  | *n=72* | *n=115* |  | *n=82* | *n=74* |  |
| HLA Mismatch |  |  | 0.320 |  |  | 0.416 |
| 0 | - | - |  | 1 (1%) | 0 (0%) |  |
| 1 | 0 (0.0%) | 2 (1.9%) |  | 1 (1%) | 1 (1%) |  |
| 2 | 0 (0.0%) | 6 (5.6%) |  | 7 (9%) | 1 (1%) |  |
| 3 | 9 (12.9%) | 11 (10.2%) |  | 12 (15%) | 9 (12%) |  |
| 4 | 16 (22.9%) | 27 (25.0%) |  | 17 (21%) | 20 (28%) |  |
| 5 | 24 (34.3%) | 36 (33.3%) |  | 31 (39%) | 30 (42%) |  |
| 6 | 21 (30.0%) | 26 (24.1%) |  | 11 (14%) | 11 (15%) |  |
| A Locus Mismatch |  |  | 0.153 |  |  | 0.239 |
| 0 | 5 (7.0%) | 4 (3.7%) |  | 7 (9%) | 6 (8%) |  |
| 1 | 24 (33.8%) | 51 (47.2%) |  | 36 (45%) | 42 (58%) |  |
| 2 | 42 (59.2%) | 53 (49.1%) |  | 37 (46%) | 24 (33%) |  |
| B Locus Mismatch |  |  | 0.218 |  |  | 0.046 |
| 0 | 0 (0.0%) | 3 (2.8%) |  | 7 (9%) | 2 (3%) |  |
| 1 | 15 (21.1%) | 30 (27.8%) |  | 24 (30%) | 13 (18%) |  |
| 2 | 56 (78.9%) | 75 (69.4%) |  | 49 (61%) | 57 (79%) |  |
| DR Locus Mismatch |  |  | 0.560 |  |  | 0.121 |
| 0 | 3 (4.3%) | 9 (8.3%) |  | 9 (11%) | 2 (3%) |  |
| 1 | 28 (40.0%) | 44 (40.7%) |  | 34 (42%) | 31 (43%) |  |
| 2 | 39 (55.7%) | 55 (50.9%) |  | 37 (46%) | 39 (54%) |  |
| **Abbreviations:** HLA, Human Leukocyte Antigen.  Data are presented as median (interquartile range) for continuous measures, and n (%) for categorical measures. | | | | | | |

| **Supplemental Table 11: Outcomes in the adult cohort subdivided into type of donor** | | | | | | |
| --- | --- | --- | --- | --- | --- | --- |
|  | **Pediatric to Adult** | | **p** | **Adult to adult** | | **p** |
|  | **Portal** | **Systemic** |  | **Portal** | **Systemic** |  |
|  | *n=72* | *n=115* |  | *n=82* | *n=74* |  |
| Graft Outcome |  |  | 0.899 |  |  | 0.095 |
| Graft Survived | 37 (51.4%) | 58 (50.4%) |  | 54 (66%) | 39 (53%) |  |
| Graft Failed | 35 (48.6%) | 57 (49.6%) |  | 28 (34%) | 35 (47%) |  |
| Recipient Outcome |  |  | 1.000 |  |  | 0.237 |
| Recipient Alive | 42 (58.3%) | 67 (58.3%) |  | 59 (72%) | 44 (59%) |  |
| Recipient Mortality | 24 (33.3%) | 37 (32.2%) |  | 19 (23%) | 26 (35%) |  |
| Lost to Follow-Up | - | - |  | 1 (1%) | 0 (0%) |  |
| Retransplanted | 6 (8.3%) | 11 (9.6%) |  | 3 (4%) | 4 (5%) |  |
| Acute Rejection till discharge |  |  | 0.405 |  |  | 0.109 |
| No | 58 (81.7%) | 88 (76.5%) |  | 60 (73%) | 62 (84%) |  |
| Yes | 13 (18.3%) | 27 (23.5%) |  | 22 (27%) | 12 (16%) |  |
| Length of Stay | 35.0 (28.0, 45.0) | 34.0 (23.0, 57.0) | 0.588 | 30.0 (20.0, 41.0) | 31.0 (21.0, 44.0) | 0.789 |
| Data are presented as median (interquartile range) for continuous measures, and n (%) for categorical measures. | | | | | | |

| **Supplemental Table 12: Multivariable Logistic regression model** **to evaluate predictors of rejection in the** **adult-to-adult cohort** | | | | |
| --- | --- | --- | --- | --- |
|  | **Odds Ratio** | **95% CI** | | **p-value** |
| Venous Drainage |  |  |  |  |
| Portal *(ref)* | --- |  |  |  |
| Systemic | 0.404 | 0.156 | 1.052 | 0.063 |
| HLA Mismatch (0-6) | 1.664 | 0.510 | 5.427 | 0.399 |
| A Locus Mismatch Level |  |  |  |  |
| 0 *(ref)* | --- |  |  |  |
| 1 | 1.171 | 0.066 | 20.847 | 0.914 |
| 2 | 1.209 | 0.029 | 50.673 | 0.921 |
| B Locus Mismatch Level |  |  |  |  |
| 0 *(ref)* | --- |  |  |  |
| 1 | 1.519 | 0.086 | 26.980 | 0.776 |
| 2 | 1.030 | 0.027 | 40.000 | 0.988 |
| DR Locus Mismatch Level |  |  |  |  |
| 0 *(ref)* | --- |  |  |  |
| 1 | 2.255 | 0.572 | 8.885 | 0.245 |
| 2 | --- |  |  |  |
| CMV High Risk |  |  |  |  |
| No *(ref)* | --- |  |  |  |
| Yes | 0.800 | 0.224 | 2.855 | 0.730 |
| Donor Age | 1.047 | 0.985 | 1.113 | 0.139 |
| Recipient Age | 0.988 | 0.953 | 1.023 | 0.494 |
| Donor Sex |  |  |  |  |
| Female *(ref)* | --- |  |  |  |
| Male | 1.738 | 0.645 | 4.683 | 0.275 |
| Recipient Sex |  |  |  |  |
| Female *(ref)* | --- |  |  |  |
| Male | 0.862 | 0.337 | 2.206 | 0.756 |
| Ethnicity of recipient |  |  |  |  |
| White | --- |  |  |  |
| African American | 2.067 | 0.514 | 8.309 | 0.307 |
| Hispanic | 4.890 | 1.060 | 22.563 | 0.042 |
| Other | --- |  |  |  |
| Total Ischemia Time | 1.317 | 1.018 | 1.705 | 0.036 |
| Serum Albumin | 1.105 | 0.515 | 2.368 | 0.798 |
| Abbreviations: CMV, Cytomegalovirus; HLA, Human Leukocyte Antigen  *Two DR mismatch omitted because of collinearity. | | | | |

| **Supplemental Table 13: Cox hazard model for adjusted graft survival analysis in the adult-to-adult cohort** | | | | |
| --- | --- | --- | --- | --- |
|  | **Hazard Ratio** | **95% Confidence Intervals** | | **p-value** |
| Venous Drainage |  |  |  |  |
| Portal *(ref)* | --- |  |  |  |
| Systemic | 0.958 | 0.525 | 1.748 | 0.889 |
| Donor Age | 1.056 | 1.016 | 1.096 | 0.005 |
| Donor Sex |  |  |  |  |
| Female *(ref)* | --- |  |  |  |
| Male | 0.942 | 0.507 | 1.750 | 0.850 |
| Donor body mass index | 1.130 | 1.019 | 1.253 | 0.021 |
| Recipient Age | 1.003 | 0.979 | 1.027 | 0.804 |
| Recipient Sex |  |  |  |  |
| Female *(ref)* | --- |  |  |  |
| Male | 0.721 | 0.385 | 1.350 | 0.307 |
| Recipient body mass index | 0.911 | 0.846 | 0.981 | 0.014 |
| Ethnicity of recipient |  |  |  |  |
| White | --- |  |  |  |
| African American | 1.834 | 0.765 | 4.393 | 0.174 |
| Hispanic | 1.409 | 0.529 | 3.750 | 0.492 |
| Other | 0.317 | 0.041 | 2.464 | 0.272 |
| Serum Albumin | 0.583 | 0.370 | 0.919 | 0.020 |
| Total Ischemia Time | 0.911 | 0.769 | 1.078 | 0.278 |
| HLA Mismatch | 1.015 | 0.808 | 1.274 | 0.901 |
| Location at Transplant |  |  |  |  |
| At home | --- |  |  |  |
| Hospitalised | 0.330 | 0.084 | 1.290 | 0.111 |
| Recent history of Septicemia |  |  |  |  |
| No | --- |  |  |  |
| Yes | 2.212 | 1.181 | 4.141 | 0.013 |
| Abbreviations: CMV, Cytomegalovirus | | | | |

| **Supplemental Table 14: Multivariable Logistic regression model** **to evaluate predictors of rejection in the** **pediatric-to-adult cohort** | | | | |
| --- | --- | --- | --- | --- |
|  | **Odds Ratio** | **95% CI** | | **p-value** |
| Venous Drainage |  |  |  |  |
| Portal *(ref)* | --- |  |  |  |
| Systemic | 1.656 | 0.695 | 3.944 | 0.255 |
| HLA Mismatch (0-6) | 0.842 | 0.341 | 2.081 | 0.710 |
| A Locus Mismatch Level |  |  |  |  |
| 0 *(ref)* | --- |  |  |  |
| 1 | 0.685 | 0.077 | 6.056 | 0.734 |
| 2 | 0.768 | 0.051 | 11.461 | 0.848 |
| B Locus Mismatch Level |  |  |  |  |
| 0 *(ref)* | --- |  |  |  |
| 1 | 0.967 | 0.042 | 22.186 | 0.983 |
| 2 | 2.166 | 0.066 | 71.538 | 0.665 |
| DR Locus Mismatch Level |  |  |  |  |
| 0 *(ref)* | --- |  |  |  |
| 1 | 1.751 | 0.601 | 5.100 | 0.304 |
| 2 | --- |  |  |  |
| CMV High Risk |  |  |  |  |
| No *(ref)* | --- |  |  |  |
| Yes | 0.335 | 0.064 | 1.758 | 0.196 |
| Donor Age | 0.972 | 0.877 | 1.076 | 0.582 |
| Recipient Age | 0.979 | 0.949 | 1.010 | 0.176 |
| Donor Sex |  |  |  |  |
| Female *(ref)* | --- |  |  |  |
| Male | 0.752 | 0.310 | 1.825 | 0.529 |
| Recipient Sex |  |  |  |  |
| Female *(ref)* | --- |  |  |  |
| Male | 2.056 | 0.859 | 4.922 | 0.106 |
| Ethnicity of recipient |  |  |  |  |
| White | --- |  |  |  |
| African American | 0.521 | 0.142 | 1.912 | 0.325 |
| Hispanic | 0.851 | 0.225 | 3.221 | 0.812 |
| Other | 10.302 | 0.866 | 122.529 | 0.065 |
| Total Ischemia Time | 0.700 | 0.525 | 0.933 | 0.015 |
| Serum Albumin | 1.350 | 0.701 | 2.598 | 0.369 |
| Abbreviations: CMV, Cytomegalovirus; HLA, Human Leukocyte Antigen  *Two DR mismatch omitted because of collinearity. | | | | |

| **Supplemental Table 15: Cox hazard model for adjusted graft survival analysis in the pediatric-to-adult cohort** | | | | |
| --- | --- | --- | --- | --- |
|  | **Hazard Ratio** | **95% Confidence Intervals** | | **p-value** |
| Venous Drainage |  |  |  |  |
| Portal *(ref)* | --- |  |  |  |
| Systemic | 0.855 | 0.527 | 1.388 | 0.526 |
| Donor Age | 0.969 | 0.917 | 1.023 | 0.255 |
| Donor Sex |  |  |  |  |
| Female *(ref)* | --- |  |  |  |
| Male | 1.521 | 0.895 | 2.584 | 0.121 |
| Donor body mass index | 0.998 | 0.931 | 1.069 | 0.956 |
| Recipient Age | 1.010 | 0.992 | 1.029 | 0.275 |
| Recipient Sex |  |  |  |  |
| Female *(ref)* | --- |  |  |  |
| Male | 1.015 | 0.615 | 1.675 | 0.954 |
| Recipient body mass index | 0.991 | 0.935 | 1.050 | 0.765 |
| Ethnicity of recipient |  |  |  |  |
| White | --- |  |  |  |
| African American | 0.721 | 0.360 | 1.444 | 0.356 |
| Hispanic | 0.800 | 0.381 | 1.682 | 0.556 |
| Other | 1.131 | 0.257 | 4.971 | 0.870 |
| Serum Albumin | 0.794 | 0.550 | 1.146 | 0.218 |
| Total Ischemia Time | 1.018 | 0.916 | 1.131 | 0.741 |
| HLA Mismatch | 1.004 | 0.830 | 1.215 | 0.964 |
| Location at Transplant |  |  |  |  |
| At home | --- |  |  |  |
| Hospitalised | 1.184 | 0.518 | 2.705 | 0.690 |
| Recent history of Septicemia |  |  |  |  |
| No | --- |  |  |  |
| Yes | 1.095 | 0.642 | 1.868 | 0.738 |
| Abbreviations: CMV, Cytomegalovirus | | | | |

| **Supplemental Table 16: Cox hazard model* for adjusted graft survival analysis in the pediatric cohort** | | | | |
| --- | --- | --- | --- | --- |
|  | **Hazard Ratio** | **95% Confidence Intervals** | | **p-value** |
| Venous Drainage |  |  |  |  |
| Portal *(ref)* | --- |  |  |  |
| Systemic | 0.597 | 0.294 | 1.209 | 0.152 |
| Donor Age | 0.884 | 0.772 | 1.012 | 0.074 |
| Donor Sex |  |  |  |  |
| Female *(ref)* | --- |  |  |  |
| Male | 0.988 | 0.494 | 1.977 | 0.972 |
| Donor body mass index | 1.006 | 0.901 | 1.123 | 0.919 |
| Recipient Age | 0.977 | 0.866 | 1.102 | 0.703 |
| Recipient Sex |  |  |  |  |
| Female *(ref)* | --- |  |  |  |
| Male | 0.753 | 0.371 | 1.526 | 0.431 |
| Recipient body mass index | 1.133 | 0.971 | 1.322 | 0.113 |
| Ethnicity of recipient |  |  |  |  |
| White | --- |  |  |  |
| African American | 1.465 | 0.632 | 3.396 | 0.374 |
| Hispanic | 1.209 | 0.451 | 3.237 | 0.706 |
| Other | 2.348 | 0.582 | 9.470 | 0.230 |
| Serum Albumin | 1.033 | 0.600 | 1.778 | 0.908 |
| Total Ischemia Time | 1.133 | 1.001 | 1.283 | 0.048 |
| HLA Mismatch | 0.758 | 0.537 | 1.070 | 0.116 |
| Location at Transplant |  |  |  |  |
| At home | --- |  |  |  |
| Hospitalised | 2.082 | 0.572 | 7.569 | 0.266 |
| Recent history of Septicemia |  |  |  |  |
| No | --- |  |  |  |
| Yes | 1.262 | 0.579 | 2.750 | 0.559 |
| Abbreviations: CMV, Cytomegalovirus  * Schoenfeld residuals testing showed that the proportional hazards assumption was met for all covariates (global p = 0.928). | | | | |

**Supplemental Figures:**


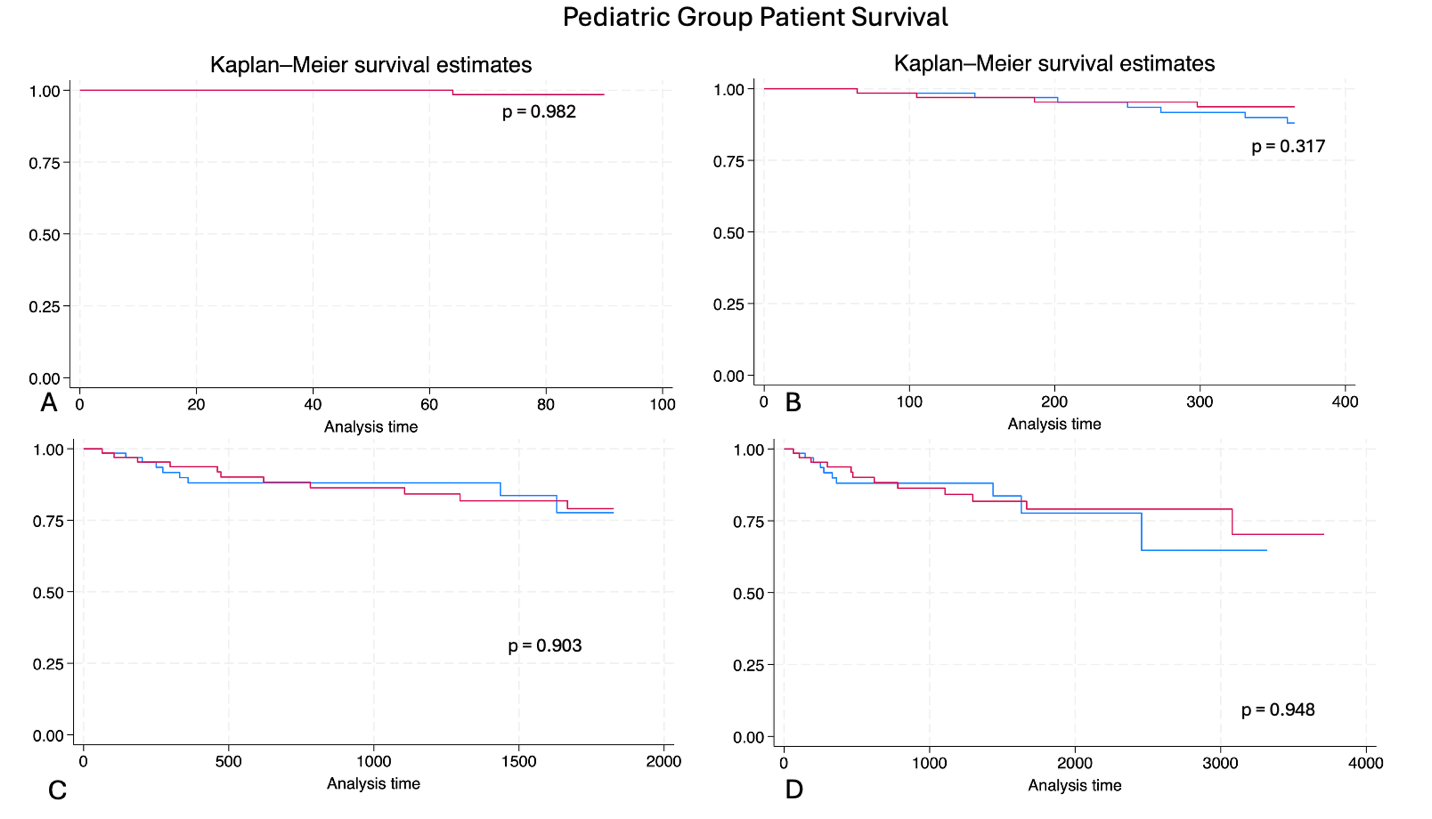
Supplemental Figure 1: Kaplan Meier curves for patient survival in the pediatric cohort at 90 days, 1 year, 5 years and 10 years

Supplemental Figure 1: 1A: Kaplan Meier curves for patient survival in the pediatric cohort at 90 days, 1B: Kaplan Meier curves for patient survival in the pediatric cohort at 1 year. 1C: Kaplan Meier curves for patient survival in the pediatric cohort at 5 years and 1D: Kaplan Meier curves for patient survival in the pediatric cohort at 10 years

Systemic Drainage

Portal Drainage


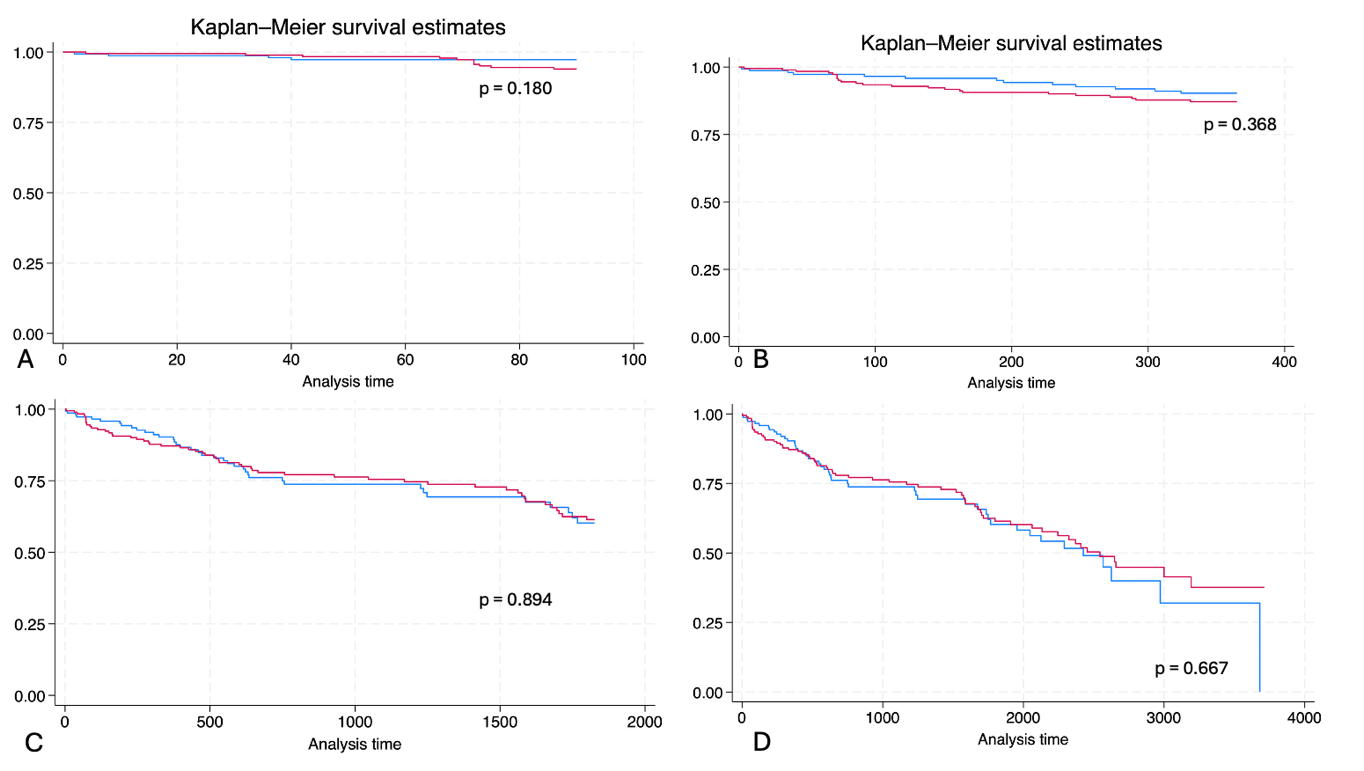
Supplemental Figure 2: Kaplan Meier curves for patient survival in the adult cohort at 90 days, 1 year, 5 years and 10 years

Systemic Drainage

Portal Drainage

Supplemental Figure 2A: Kaplan Meier curves for patient survival in the adult cohort at 90 days, 2B: Kaplan Meier curves for patient survival in the adult cohort at 1 year. 2C: Kaplan Meier curves for patient survival in the adult cohort at 5 years and 2D: Kaplan Meier curves for patient survival in the adult cohort at 10 years
